# Rethinking Termite Methane Emissions: Does the Mound Environment Matter?

**DOI:** 10.1111/gcb.70838

**Published:** 2026-04-09

**Authors:** Abbey R. Yatsko, Paul Eggleton, Caleb Jones, Marcos Pérez‐Losada, Ignacio Ramos‐Tapia, Jeff R. Powell, Baptiste Wijas, Amy E. Zanne

**Affiliations:** ^1^ Biology Department University of Miami Coral Gables Florida USA; ^2^ Life Sciences Department The Natural History Museum London UK; ^3^ ArborMeta Byron Bay New South Wales Australia; ^4^ Milken Institute School of Public Health The George Washington University Washington DC USA; ^5^ Center for Bioinformatics and Integrative Biology, Facultad de Ciencias de la Vida Universidad Andres Bello Santiago Chile; ^6^ Hawkesbury Institute for the Environment Western Sydney University Penrith New South Wales Australia; ^7^ Cary Institute of Ecosystem Studies Millbrook New York USA

**Keywords:** Australian savanna, carbon cycle, methane emissions, temperature, termite, termite emission factor, termite mound structure

## Abstract

Termites are important decomposers in tropical ecosystems and emit methane (CH_4_) as they digest plant material. Global estimates of termite‐derived CH_4_ are calculated using termite emission factors (TEF, measured from individuals) and estimated biomass. However, this approach overlooks how the termite mound, via internal and external factors, may influence emissions to the atmosphere. Termite feeding habits, mound methanotrophs and mound structure (internal environment), as well as temperature and season (external environment) can influence net CH_4_ emission but remain unparameterized. We investigated how these factors shaped CH_4_ emissions from three dominant mound‐building termite species *(Coptotermes acinaciformis*, *Nasutitermes magnus*, and *Amitermes laurensis*) in a northern Australian savanna across four seasons. We compared species‐level TEFs and emissions at the mound‐ and landscape‐scales to evaluate relative species contributions, both with and without accounting for the internal and external environment. We hypothesized that larger, thinner‐walled mounds would emit greater CH_4_, and that emissions would be higher at high temperatures and during wet seasons. We expected greater emissions with lower abundances of methanotrophs and *pmoA* gene copies (involved in CH_4_ oxidation) in mound material. *Coptotermes acinaciformis* individuals had the highest TEFs (1.07 μg CH_4_ g^−1^ termite h^−1^), *N. magnus* mounds emitted the most CH_4_ (3426 μg CH_4_ h^−1^ m^−2^) and *A. laurensis* had the highest emissions at the landscape scale (1.04 × 10^−9^ Tg CH_4_ ha^−1^ year^−1^). CH_4_ emissions increased with temperature and were highest in the wet‐to‐dry transition season. Mound structure, bacterial methanotroph communities, and *pmoA* abundance had no effect on CH_4_ emissions. Our results highlight the limitations of relying solely on TEFs to estimate contributions of termites to global CH_4_ emissions and emphasize the importance of incorporating external environmental conditions, while further exploring internal mound processes. This information allows more accurate parameterization of termite CH_4_ contributions to savanna carbon and global CH_4_ budgets.

## Graphical abstract

Many factors can influence the amount of methane (CH_4_) that is released from a termite mound. In this study, we tested how the external and internal environment of a termite mound impacts CH_4_ emission using field measurements of mounds in a Northern Australia savanna. We explored external environmental factors, such as temperature and season, as well as internal factors, such as termite feeding habits, mound structure, and methanotrophic microbial communities. Ultimately, we found that external environmental factors played a stronger role in predicting CH_4_ emissions, and this information can be used in upscaling the impact of termite CH_4_ emissions at global scales.

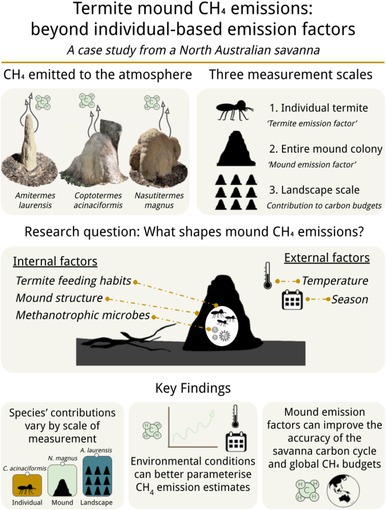

## 1 Introduction

Termites are key decomposers, especially in the tropics, dominating terrestrial arthropod biomass at an estimated global biomass of 100 Mt. (Rosenberg et al. 2023) and cycling significant quantities of terrestrial carbon (Seibold et al. 2021; Griffiths et al. 2019). As a by‐product of carrying out decomposition, termites release carbon dioxide (CO_2_), but also methane (CH_4_). CH_4_ is produced by symbiotic methanogens (Archaea) in termite guts as they break down wood, organic matter in soils, and dead plant matter (Zimmerman et al. 1982; Brune and Dietrich 2015). CH_4_ is a powerful greenhouse gas that contributes to climate warming (IPCC 2021), but there exist many uncertainties in quantifying natural CH_4_ sources (i.e., emissions) and sinks (i.e., removal, conversion, or storage) that comprise the CH_4_ budget (Kirschke et al. 2013; Saunois et al. 2020). Termite CH_4_ is one such poorly parameterized component of the global CH_4_ budget (Jamali, Livesley, Grover, Dawes, et al. 2011; Khalil et al. 1990) current estimates show that termites emit 8–21 Tg CH_4_ per year (Ito 2023). However, this value is highly debated (Zimmerman et al. 1982; Saunois et al. 2020), since variation in termite ecology is not captured in present estimates (Law, Allison, et al. 2024). To resolve such uncertainties, a more nuanced understanding of the underlying factors contributing to termite CH_4_ emissions is required (Law, Allison, et al. 2024).

Estimates for global termite CH_4_ emissions are typically derived by multiplying a termite emission factor (TEF: average CH_4_ emission per termite biomass, μg CH_4_ g^−1^ termite h^−1^) by regional‐scale estimates of termite biomass (Khalil et al. 1990; Ito 2023; Saunois et al. 2020). Species variation in TEFs spans a wide range, from negligible emissions to more than 25 μg CH_4_ g^−1^ termite h^−1^, which is largely attributed to differences in feeding groups (Sanderson 1996; Zhou et al. 2022) and termite gut microbial composition (i.e., the presence of CH_4_‐producing Archaea, Rouland et al. 1993). It has been shown that soil feeders (*Cubitermes* sp.) and grass feeders (*Trinervitermes* sp.) have high TEFs (Sanderson 1996; Zhou et al. 2022), potentially due to higher archaeal‐to‐bacterial ratios in their guts (Rouland et al. 1993; Arora et al. 2022). Additionally, accurately estimating global termite biomass is a challenging venture, due to the diverse sizes and constructions of termite colonies, including epigeal (above ground) mounds, subterranean colonies, arboreal nests, and colonies in deadwood (Law, Allison, et al. 2024).

Termite contributions to global CH_4_ emissions are therefore estimated using an overly simplistic model that fails to capture the complexities of how termite ecology influences net CH_4_ emission to the atmosphere (Law, Allison, et al. 2024). This failure is especially true when considering mound‐building termites, which are important decomposers of wood, soil, plant litter, and grass, particularly in savanna ecosystems (Zanne et al. 2022; Bunney et al. 2024). Tropical savannas often feature large epigeal termite mounds (in Africa: Meyer et al. 1999; Davies et al. 2014, in Australia: D'hont et al. 2021, in South America: Neto et al. 1986, in India: Jouquet et al. 2015), sometimes covering up to 5%–7% of the land area (Levick et al. 2010; Holdo and McDowell 2004). In this way, termite mounds represent a concentration of termite biomass and CH_4_ emission (Jamali, Livesley, Dawes, Cook, et al. 2011; Brümmer et al. 2009; Van Asperen et al. 2021; Jamali, Livesley, Grover, Dawes, et al. 2011), which can overcome the oxidative capacity of soils, which are recognized as CH_4_ sinks (Eggleton et al. 1999; Dunfield 2007). However, current CH_4_ calculations fail to consider mounds at all, and it is therefore necessary to determine how internal components of the termite mound (structure, mound microbial communities) and local external environmental conditions shape mound CH_4_ release.

Structural traits of termite mounds define the internal mound environment and shape the capacity for internal gas exchange and diffusion to the atmosphere (Ocko et al. 2019). Wall thickness is one such trait that varies widely across species and locations (Korb 2011). Greater wall thickness, while more protective from the outside environment and organisms trying to access the mound, can limit diffusion and result in an accumulation of gases, such as CO_2_, inside the mound (Korb 2003). Further, when termite mounds grow, they can support a larger termite population (Josens and Soki 2010), with the potential to generate higher total mound CH_4_ emissions (Van Asperen et al. 2021; Jamali, Livesley, Dawes, Hutley, and Arndt 2011; Vesala et al. 2023). Therefore, identifying how wall thickness and mound size influence CH_4_ emission will provide insight on the importance of incorporating these factors in CH_4_ upscaling efforts.

Additionally, external local environmental conditions such as temperature and season influence termite activity and CH_4_ emissions. Termite wood decomposition rates are highly sensitive to increasing temperatures, which could result in increased CH_4_ emissions (Zanne et al. 2022; Law, Flores‐Moreno, et al. 2024). Direct relationships between termite CH_4_ emission, decomposition activity, and temperature remain unexamined, but previous research suggests that mound CH_4_ emissions are highest at the warmest part of the day (Jamali, Livesley, Dawes, Cook, et al. 2011; Räsänen et al. 2023). Seasonality has also been shown to affect termite mound CH_4_ emissions: previous research in an Australian tropical savanna showed significantly higher mound CH_4_ emission during the wet season than for other times of the year (Jamali, Livesley, Dawes, Cook, et al. 2011), which could be due to changes in termite biomass (Jamali, Livesley, Dawes, Hutley, and Arndt 2011) or temperature. However, other studies found mound emissions to be highest in the dry season (Quevedo et al. 2021; Räsänen et al. 2023), which could result from decreased oxidation of methane by microbial activity resulting from moisture limitations. Determining the role of temperature and seasonality in termite mound CH_4_ emissions is therefore necessary for accurate annual CH_4_ emission estimates.

Finally, CH_4_ released from individual termites residing within a mound may not reach the atmosphere due to the presence of methanotrophic (CH_4_‐consuming) bacteria and archaea within termite mounds. Methanotrophic bacteria have been shown to reduce the net emission of CH_4_ from the mound to the atmosphere by 50% (Nauer, Hutley, and Arndt 2018). The mound microbial community can therefore influence net CH_4_ emission, which is known to vary across termite species (Ho et al. 2013; Chiri et al. 2020). The gene encoding the particulate methane monooxygenase subunit A (*pmoA*) is involved in the CH_4_ oxidation pathway for most bacterial methanotrophs (Cupples and Thelusmond 2022). The *pmoA* gene plays an important role in CH_4_ metabolism, and its abundance in termite mound material is a standard method for identifying methanotrophic microbe communities (Chiri et al. 2020). However, direct associations between methanotroph community composition, *pmoA* gene abundance, and termite mound CH_4_ emission are yet to be tested.

In this study, our goal was to refine our understanding of termite contributions to CH_4_ emissions by parsing out various factors that influence net mound CH_4_ emission to the atmosphere. To do so, we combined previous measures of mound abundances (Clement et al. 2021) with new measures of species‐level TEFs and mound‐level CH_4_ emissions from three dominant mound‐building termite species, with varied feeding habits and mound traits, in North Queensland, Australia tropical savannas. We asked the following questions: 1. How do species‐level TEFs contrast with average mound‐level CH_4_ emissions and landscape‐scale CH_4_ emissions? 2. What is the relative influence of species‐specific mound structural traits (mound size and wall thickness) and external environmental drivers (temperature and season) on mound‐level CH_4_ emissions? 3. Do methanotrophic microbial communities (archaea and bacteria) and methanotroph gene (*pmoA*) abundance in the mound material influence CH_4_ emission within and across termite mounds?

## 2 Methods

### 2.1 Study Site

**FIGURE 1:**
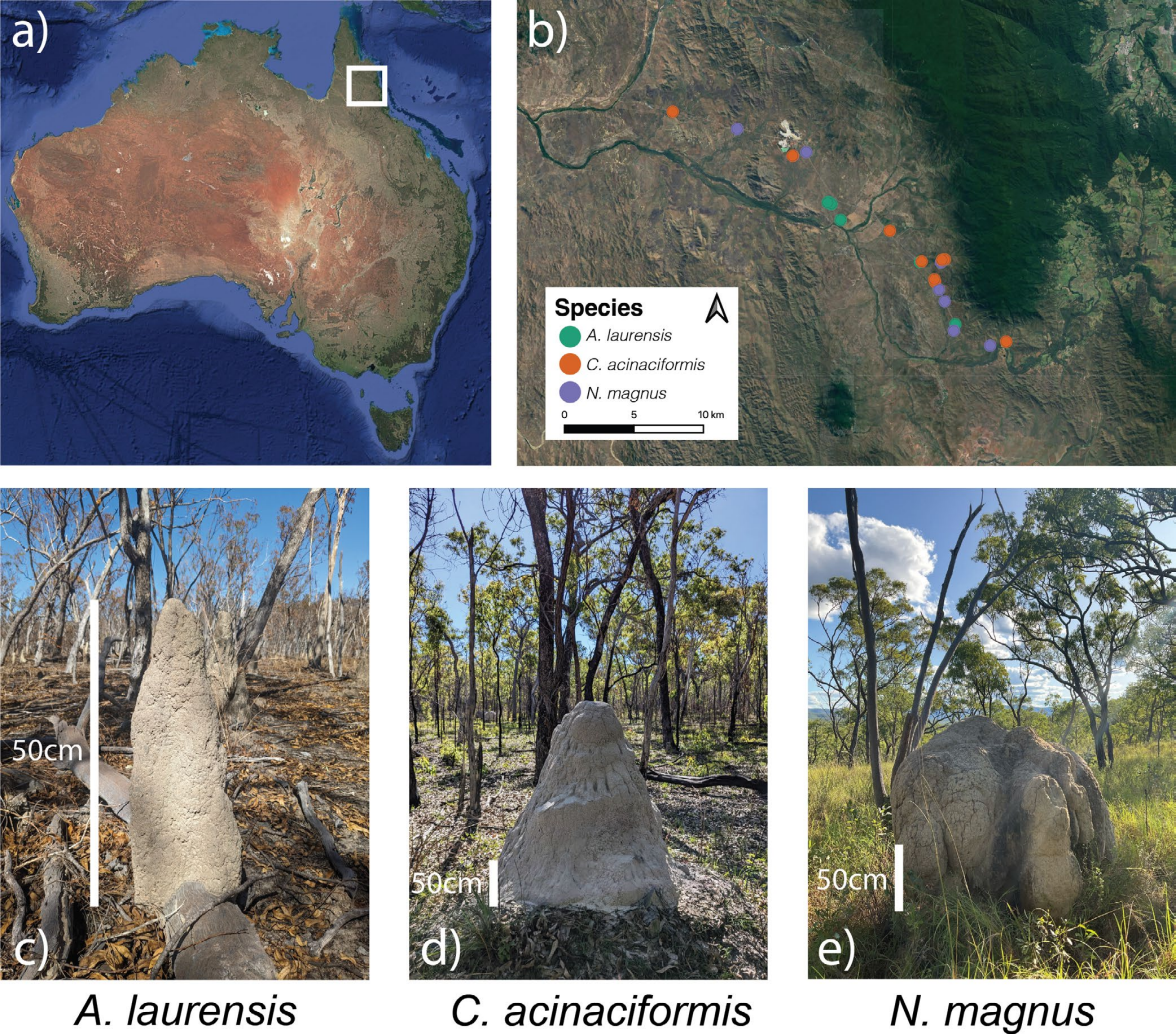
(a) Study location in far North Queensland, Australia (white square). (b) Location of sampled termite mounds repeatedly measured across all four seasons (colored by species, *n* = 19 total, *n* = 7 *A. laurensis*, *n* = 7 *C. acinaciformis* , *n* = 5 *N. magnus* ) in the greater Station Creek region. Termite mound morphologies common to (c) *A. laurensis*, (d) *C. acinaciformis* , (e) *N. magnus* . White scale bars on each species mound morphology image indicate a reference height measurement of 50 cm. Mean dimensions of each species' mound can be found in Table S13.

Termite mounds were sampled at the Australian Wildlife Conservancy (AWC) Brooklyn Sanctuary in the Station Creek region (−16.61 S, 145.24 E, Figure 1). The Station Creek region comprises a wet savanna ecosystem with distinct wet and dry seasons, experiencing 1728 mm of rainfall annually (Cheesman et al. 2018). Floristically, the region is dominated by *Eucalyptus cullenii*, *Corymbia clarksoniana, Acacia disparrima* subsp. *Calidestris*, and *Larsenaikia ochreata* (Flores‐Moreno et al. 2024). Termite diversity and abundance in this region are relatively high for savanna ecosystems, with 14 species described to occur in the area (Clement et al. 2021). *Amitermes laurensis*, *Coptotermes acinaciformis*, and *Nasutitermes magnus* are abundant mound‐building termite species (Clement et al. 2021) and were selected as the focal species of this study. *Coptotermes acinaciformis* feeds on wood and typically builds medium to large‐sized mounds with a dome shape (mean height = 87 cm, Clement et al., unpublished data), which are often built at the base of trees which they frequently hollow (Yatsko et al. 2024, 2025). *Nasutitermes magnus* feeds on grass and builds large mounds (mean height = 92 cm, Clement et al. unpublished data). *Amitermes laurensis* feeds on plant litter and builds small conical mounds (mean height = 59 cm, Clement et al. unpublished data) that have high abundance in the landscape (see Figure 1 for mound morphologies).

### 2.2 Determining Species‐Level Termite Emission Factor With Laboratory Measurements

In February 2024 we collected mound material from the field and conducted laboratory measurements to calculate a TEF for each species. To do so we removed entire *A. laurensis* mounds (*n* = 5) from the field due to their small size. For *C. acinaciformis* (*n* = 5) and *N. magnus* (*n* = 5), mounds were too large, so we subsampled mound pieces using a saw and chisel. Mound samples were placed in a bucket and transported back to laboratory conditions where they equilibrated to room temperature (24°C) for a minimum of 12 and a maximum of 24 h prior to CH_4_ emission measurement. We cut mound samples into smaller pieces (approximately 5 × 5 cm) and removed all termites from the mound piece using entomological forceps (mean termite biomass = 2.2 g) to measure CH_4_ emissions directly from termite individuals. CH_4_ emissions were measured from combined samples of worker and soldier termites. We note that each species had a different ratio of workers to soldiers (*A. laurensis*: 99% workers, 1% soldiers. *N. magnus* : 75% workers, 25% soldiers. *C. acinaciformis* : 56% workers, 42% soldiers). We measured CH_4_ emissions using a Los Gatos Research (LGR) Ultraportable Greenhouse Gas Analyzer (UGGA, San Jose, CA, United States) connected to a closed sampling chamber made from PVC and plexiglass (LI‐COR 8200–104, Lincoln, Nebraska, United States) with a volume of 1570.8 cm^3^. Measurements were taken over the course of 300 s following a 60 s calibration (Figure S3). We divided emissions by termite biomass (g, wet mass) to calculate the species‐level TEF, defined as mean μg CH_4_ g termite^−1^ h^−1^ (Zhou et al. 2022).

### 2.3 Termite Mound CH_4_ Emission Field Sampling and Species Identification

Termite mound CH_4_ emissions were sampled in the field (separate from laboratory measurements described above) at four times between May 2022 and February 2024 to capture the range of seasonal conditions and temperatures characterizing the savanna ecosystem (Table S1). We selected mounds for each of the three termite species based on the following criteria: (a) mounds were accessible by vehicle across weather conditions, (b) mounds were active and occupied by termites at the time of measurement, (c) mounds were representative of the range of sizes for a given species, and (d) mounds were in locations with a low likelihood of human interference. The location of each mound was recorded with GPS coordinates.

The initial survey in May 2022 (wet‐to‐dry transition season) targeted a larger number of mounds per species (*A. laurensis*: *n* = 14, *C. acinaciformis* : *n* = 25, *N. magnus* : *n* = 21, Table S2). Then, eight mounds from each species were randomly selected from the May 2022 campaign for remeasurement in November 2022 (dry‐to‐wet season transition), August 2023 (mid‐dry season), and February 2024 (mid‐wet season). Over the course of sampling, there were five instances where mounds died or were destroyed and could not be re‐measured (Table S2). When this was the case, the mound was replaced with a mound closest in size from the original May 2022 sampling pool. In February 2024, we were only able to re‐measure seven *N. magnus* mounds due to logistical constraints of accessing mounds following Cyclone Jasper which affected the region in December 2023. At the end of the experiment, seven *A. laurensis*, seven *C. acinaciformis*, and five *N. magnus* mounds were re‐measured during all four seasons (Table S2, Figure 1). These mounds were used to analyze the effect of season and temperature on mound CH_4_ emission.

Two soldiers and five workers were sampled from each mound in the May 2022 campaign and stored in 95% ethanol. Soldier sample morphology was used to confirm species‐level identification (P. Eggleton, personal communication, May 2023). When visual identification was not possible, we followed the protocol in Clement et al. (2021) for species identification using DNA barcoding of the COII gene.

### 2.4 Field‐Based Measurements of Termite Mound CH_4_ Emissions

We sampled CH_4_ emissions from termite mounds in the field using a semi‐closed chamber‐based sampling system connected to the LGR UGGA (Figure S1). For each mound, 4–5 surface measurements were taken by attaching the LGR UGGA to a semi‐closed PVC sampling chamber (Jeffrey et al. 2020) via gas tubing. Sampling chambers were placed on the mound surface without breaking the mound wall (Figure S1a) and secured with an external airtight seal using a ring of inert potting clay (Walker Ceramics, Bayswater, VIC, Australia; Jeffrey et al. 2020, Figure S1b).

Sampling chambers were fixed on the flattest surface of the mound to accurately calculate chamber volume which was approximated as a cylinder. Different sized chambers were used for the three species, as *A. laurensis* mounds were smaller (chamber volume = 2960.1 cm^3^, surface area = 183.9 cm^2^) and *C. acinaciformis* and *N. magnus* mounds could fit medium chambers (chamber volume = 899.9 cm^3^, surface area = 83.3 cm^2^). We standardized the mound sampling points to optimize for the unique geometries of each mound. *Coptotermes acinaciformis* and *N. magnus* mounds were sampled in five locations: north‐facing side at 50% mound height, east‐facing side on the flattest surface closest to the mound base, south‐facing side at 50% mound height, west‐facing side on the flattest surface closest to the base, and the top of the mound at the flattest surface (Figure S1c). Due to their conical shape, *A. laurensis* mounds were sampled in the first four locations but with the top measurement omitted, since there was no flat surface available. Individual sampling points were marked so that the sampling position could be repeated during remeasurement.

After chambers were fixed to the mound and sealed with clay, CH_4_ emission was recorded for 120 s and internal chamber air temperature (°C, temperature at the mound surface) was simultaneously recorded every minute using a thermocouple probe (PerfectPrime TC41, 4‐Channel K‐Type Digital Thermometer Thermocouple Sensor). All measurements were made between 08:00 and 17:00. Measurement quality assessed using two criteria: (1) no humming noise coming from the sampling chamber, which indicated that the clay ring seal was airtight and free of leaks (Jeffrey et al. 2020), and (2) real‐time measurements on the LGR UGGA displayed positive, linear slopes, indicating constant production of CH_4_ (indicating termite occupation) and no leaks. Following CH_4_ emission measurement, a small area of the mound was broken to access galleries and confirm termite occupation.

### 2.5 CH_4_ Emission Calculation

CH_4_ emissions from termite mounds and individual termites (μg CH_4_ h^−1^) were calculated using linear regression slopes (x = time, y = [CH_4_] in chamber headspace) following the ideal gas law and using average chamber air temperature for each measurement and assuming ambient pressure. *R* ^2^ and *p*‐values were calculated for each measurement, and samples with *R* ^2^ < 0.4 were manually inspected for erroneous points. Erroneous points often were present at the beginning or end of the measurement due to the chamber opening or closing and were omitted when present.

Positive slopes indicated net CH_4_ emission, while negative slopes indicated that CH_4_ oxidation exceeded production. In our data collection and analysis, we only considered termite mounds to be alive if the average mound emission was a positive value. However, within an individual mound it was possible for some subsamples to show negative slopes, indicating net CH_4_ oxidation. For analyses where emissions from subsampled areas of the mound were the unit of measure (i.e., mound microbial communities, see below), negative values for CH_4_ emission were included in the analysis. For analyses run at the mound‐level (i.e., structural equation modeling, see analyses below), we only considered live mounds and therefore CH_4_ emission was always positive. CH_4_ emissions for each subsample were divided by the surface area covered by the sampling chamber to derive emissions per unit mound surface area (μg CH_4_ h^−1^ m^−2^). We assessed variation in emissions within mounds by calculating a Coefficient of Variation (CV, Table S12), but as our second research question was not focused on intra‐mound variation, we used a mound‐level average in subsequent analyses. Total mound emission was derived by multiplying average mound emission per unit mound surface area by total surface area of the mound (μg CH_4_ h^−1^ mound^−1^).

### 2.6 Mound Size and Wall Thickness Measurement

We used photogrammetry to estimate mound surface area and volume. Photogrammetry uses multiple photographs taken from different angles to construct a digital three‐dimensional model of the scanned object (Nauer, Chiri, et al. 2018). To take photogrammetry measurements we first removed debris surrounding the mound and matted down any grass obscuring the mound. Starting at the north‐facing side of the mound, consecutive photos were taken on an Apple iPad Pro (Cupertino, California, United States) as the person scanning walked clockwise around the mound, holding the iPad steady. The top of the mound was also scanned when it was possible; on occasion this was constrained by mound height. Photos were digitally assembled in the Polycam app (Polycam Inc.) to create a three‐dimensional photogrammetric termite mound model.

Individual mound models were exported as .glb files from Polycam app and imported into MeshLab to remove non‐termite mound material using the selection tool. Total mound surface area was calculated in MeshLab using the *Compute Geometric Measures* function. To calculate total mound volume, cleaned mound models were imported into Blender as. obj files (Blender Online Community 2022). Each mound was first filled with the *grid fill* command, and volume was calculated using the 3D print toolbox *volume* function. One limitation of the mound volume estimation method is that we were not directly able to quantify the termite biomass within each mound.

Mound wall thickness was recorded at the final measurement campaign (February 2024) due to the invasive nature of the measurement. After sampling CH_4_ emissions, we used a drywall hammer or pickaxe (depending on mound hardness) to break the mound wall at each sampling point. We used digital calipers with 0.01 mm precision to record wall thickness, which was defined as the distance from the external wall to where termite galleries were visible.

### 2.7 Mound CH_4_ Emissions Estimated at the Landscape Scale

We used data from Clement et al. (2021) which reports landscape abundance of occupied termite mounds per hectare for the three study species and at the Station Creek site. We calculated average annual mound CH_4_ emission for each of our study species (Tg CH_4_ year^−1^ mound^−1^) and then multiplied this value by mound density per hectare (Clement et al. 2021) to derive an estimate of landscape‐scale termite mound emissions for northern Australian savanna ecosystems (Tg CH_4_ year^−1^ ha^−1^). It is estimated that tropical savannas in northern Australia cover approximately 1.9 million km^2^ (Chen et al. 2003), and termite mounds are characteristic features of this landscape, although it is noted that the proportion of dominant species may change for different locations in Australian tropical savannas.

### 2.8 Mound Microbial Community Sampling and Metagenomic Sequencing

In May 2022 we sampled termite mound material to link microbial communities directly with CH_4_ emissions. Following CH_4_ emission measurement, the area covered by the chamber was broken to sample internal mound material (where termite galleries were, Chiri et al. 2020) using a sterilized chisel and forceps. Mound substrate was placed into a 5 mL soil vial and subsequently frozen at −20°C.

We selected a subset of samples for microbial community analysis based on their suitability to address two questions: is microbial community variation associated with variation in CH_4_ emission (1) between mounds and (2) within mounds? To test drivers of variation between mounds, for each of the three study species we selected the four mounds with the highest average CH_4_ emission and the four with the lowest average CH_4_ emission. For these mounds (*n* = 8 per species, *n* = 24 total mounds), we selected three subsamples within each mound that were closest to the average CH_4_ emission for that mound for metagenomic sequencing (*n* = 72 total subsamples). To test drivers of variation within mounds, we first calculated the CH_4_ emission range for each mound and then identified the five mounds with the highest variation for each species, indicated by the greatest CH_4_ emission range. For these mounds we selected all subsamples (4 or 5) collected (*n* = 5 mounds per species, = 15 total mounds, *n* = 69 total subsamples). In total, we extracted DNA from and sequenced 115 mound samples, and there was overlap in which some samples were considered for both questions (i.e., a highly variable mound could also be a top‐emitting mound).

DNA extractions were carried out with the DNeasy PowerSoil Pro Kit (Qiagen, United States) using the manufacturer's instructions with an input of approximately 250 mg mound material. Mound material was homogenized prior to extraction using a FastPrep‐24 5G Homogenizer MP Biomedicals (MP Biomedicals, United States; settings: sediment—soils/rocks, Speed: 5.5 m s^−1^, Adapter: quickpro, Time: 30 s, Lysing matrix: E, Quantity: 50 mg, Cycles: 2, Pause time: 300 s). DNA concentration (ng μL^−1^) and quality (260/280 and 260/230 values) were evaluated with a NanoDrop spectrophotometer (Thermo Fisher Scientific, Massachusetts, United States).

Metagenomic sequencing was performed at the Ramaciotti Centre for Genomics (UNSW, Sydney, Australia) on a NovaSeq X Plus 10B (2 × 150 bp; Illumina). Raw reads were first quality controlled using fastp v0.23.4 (Chen et al. 2018), then go reads error correction using the Bayesian‐Hammer as implemented in SPAdes v4.0.0 (Prjibelski et al. 2020). Resultant corrected reads were assembled using Megahit v1.2.9 (Li et al. 2016); only contigs over 1 kb were retained for gene prediction. Parallelized version of Prodigal—pprodigal (Hyatt et al. 2010; Jaenicke 2022) was used for gene prediction; nonredundant genes (protein translations) were generated using CD‐HIT v4.8.1 (Li and Godzik 2006), with parameters setting as ‐c 0.9 ‐M 0 ‐T 0 ‐l 20 ‐G 0 ‐aS 0.9 ‐g 0. KEGG Orthology (KO) and pathway analysis was performed using KofamKOALA (Aramaki et al. 2020 KofamScan v1.3.0 with the latest KOfam database v2024‐08‐29). Resultant tables (reads count per sample) including KO and pathway analysis were generated using CoverM (Aroney et al. 2024).

### 2.9 Identifying Methanotrophic Microbes and Genes From Metagenomic Sequencing

From the metagenomic analyses described above, we targeted the *pmoA* gene as it plays a direct role in CH_4_ metabolism (Zhou et al. 2015) and has been previously associated with termite mound CH_4_ oxidation (Chiri et al. 2020). We removed five samples with low total read counts (< 300 reads) compared to the average read count across the dataset (mean = 18,762,769 reads). To account for variation in sequencing depth, we then resampled reads for the *pmoA* gene (KO number “K10944”) to standardize total reads to the minimum value of the dataset (6,913,446 reads). We calculated the relative abundance of the *pmoA* gene to use in subsequent analyses.

We identified methanotrophic bacterial genera from various literature sources and then queried our metagenomic sequencing data outputs for these 16 genera (Table S11). From this list, 10 methanotroph genera were present in our mound samples. Regarding methanotrophic archaea, these genera are less well described in comparison to bacteria, although the *Candidatus methanoperedens* genus is known to be associated with methanotrophy (Bhattarai et al. 2019). However, we did not detect any methanotrophic members in the archaea metagenomic dataset, and therefore focused our analyses on the bacterial methanotroph community composition at the genus level.

### 2.10 Analyses

#### 2.10.1 CH_4_ Emissions at the Individual Termite and Mound‐Level

We used Analysis of Variance (ANOVA) to evaluate differences in TEFs of the three termite species. We used the R package *emmeans* (Lenth 2025) to perform pairwise species comparisons with Tukey's HSD adjustment for multiple testing. To evaluate differences among species in termite mound CH_4_ emission, we used a linear mixed effect model from the R package *lmer* (Bates et al. 2015). In the model, CH_4_ emission was the response, species was a fixed effect, and mound individual was included as a random effect to account for multiple measurements for each mound. In a post hoc analysis, we compared estimated marginal means among species using Tukey's HSD adjustment for multiple comparisons.

#### 2.10.2 Influence of Species‐Specific Mound Structural Traits and External Environmental Drivers on Mound‐Level CH_4_ Emission

We used piecewise Structural Equation Modeling (SEM) to explore the direct and indirect drivers of mound‐level CH_4_ emission from termite mounds. Specifically, we modeled how mound volume and mean wall thickness mediated the relationship between termite species and CH_4_ flux, while also accounting for temperature and season effects. The SEM was built using the following three equations: (1) Mound volume (V) as a function of species (SP) in a linear model, (2) Mean wall thickness (W) as a function of species in a linear model, and (3) Mean CH_4_ emission (E) as a function of all predictors (mean wall thickness (W), volume (V), species (SP), temperature (T), and season (S)) in a random effects model with individual mound as the random effect:

(1) $$V=\beta_{0,V}+\beta_{1,V}\times \mathrm{SP}+\varepsilon_{V}$$

(2) $$W=\beta_{0,W}+\beta_{1,W}\times \mathrm{SP}+\varepsilon_{W}$$

(3) $$E=\beta_{0,E}+\left(\beta_{1,E}\times V\right)+\left(\beta_{2,E}\times W\right)+\left(\beta_{3,E}\times \mathrm{SP}\right)+\left(\beta_{4,E}\times T\right)+\left(\beta_{5,E}\times S\right)+\varepsilon_{E}$$

where *β*_0,*x*_ is the model intercept for a given predictor variable (*x*), *β*_*x*_ is the slope of each predictor variable, and *ε*_*y*_ is the error of the response variable (*y*). In this analysis, mound volume and mean wall thickness predictors were scaled (mean‐centered and divided by standard deviation).

The SEM was built using the *psem* function from the *piecewiseSEM* R package (Lefcheck 2016). We assessed model fit using the Fisher's C statistic, and directed separation tests were used to verify that there were no important missing paths. Individual component models in the SEM were evaluated using standardized coefficients, confidence intervals, and *p*‐values.

### 2.11 Mound Methanotroph Communities, Genes, and Influence on CH_4_ Emission: Variation Between Mounds

For the subset of metagenomic data that captured variation between mounds, we explored patterns in the composition of methanotroph genera within termite mounds using a Principal Coordinates Analysis (PCoA). The analysis was based on a Bray–Curtis dissimilarity matrix of the mound methanotroph community and used the *wcmdscale* function from the *vegan* R package (Oksanen et al. 2025) with 2 dimensions (*k* = 2). We calculated weighted genera scores, and the resulting PCoA1 and PCoA2 scores were used to explore relationships between methanotroph community structure, termite species, and variation in CH_4_ emission.

We then tested if methanotroph communities were predicted by termite species or by CH_4_ emission using permutational multivariate analysis of variance (PERMANOVA) with the *adonis2* function (Oksanen et al. 2025), including 999 permutations to assess significance. Then, we used a linear mixed effect model to determine the relationship between methanotroph communities (summarized by PCoA1 and PCoA2 scores) and the relative abundance of the *pmoA* gene. In the model, PCoA1, PCoA2, and their interaction were predictors, and *pmoA* relative abundance was the response. Termite mound individual was included as a random effect.

Finally, we used a linear mixed effect model to determine if methanotroph community and gene predictors influenced CH_4_ emission. Model predictors included the relative abundance of the *pmoA* gene, termite mound species, the interaction between PCoA1 and PCoA2 scores, and the sum of all methanotroph genera relative abundances. Mound individual was included as a random effect. To explore this same relationship for each individual methanotroph genus, we ran separate models for each genus where the relative abundance of an individual methanotroph genus and termite mound species were predictors, CH_4_ emission was the response, and mound individual was a random effect.

### 2.12 Mound Methanotroph Communities, Genes, and Influence on CH_4_ Emission: Variation Within Mounds

For the subset of metagenomic data that captured variation within mounds, we carried out the same PCoA as described above to test PCoA1 and PCoA2 scores (including their interaction) as well as relative abundance of the *pmoA* gene, termite mound species, and the relative abundance of the sum of all methanotroph genera as predictors of CH_4_ emission. Mound individual was included as a random effect. All analyses were conducted in R version 4.3.2.

## 3 Results

### 3.1 Scaling CH_4_ Emissions From Individual Termite‐ to Mound‐ to Landscape‐Levels

**FIGURE 2:**
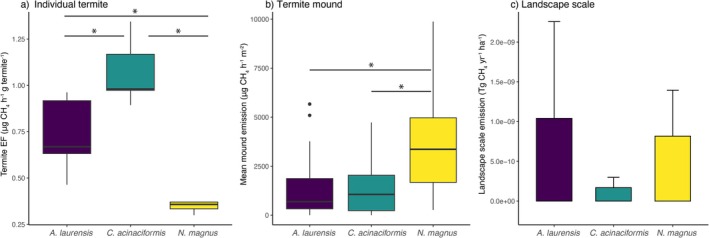
(a) Termite emission factors (μg CH_4_ h^−1^ g termite^−1^) for the study species (purple = *A. laurensis*, green = *C. acinaciformis* , yellow = *N. magnus* ). (b) Average mound‐level CH_4_ emission by mound surface area (μg CH_4_ h^−1^ m^−2^) for the three study species. Boxplot center lines indicate the median and whiskers show ±1.5 IQR, and asterisks indicate significantly different pairwise contrasts. (c) Bar plot of landscape‐scale mound CH_4_ emissions (Tg CH_4_ year^−1^ ha^−1^) by species. Bar plot error bars indicate one standard deviation.

TEF values for individual termites (μg CH_4_ g^−1^ termite h^−1^) varied significantly by species (*F*_(2,12)_ = 29.41, *p* < 0.001) with *C. acinaciformis* (mean TEF = 1.07) having a higher TEF than *A. laurensis* (mean TEF = 0.73), which had a higher TEF than *N. magnus* (mean TEF = 0.37). All pairwise comparisons were significant (Figure 2a, Table S3). Average CH_4_ emissions from termite mounds (per unit surface area) also significantly varied by species (*F*_(2,125)_ = 16.74, *p* < 0.001) where *N. magnus* (3426 μg CH_4_ h^−1^ m^−2^) had significantly greater emissions than both *A. laurensis* (1270 μg CH_4_ h^−1^ m^−2^) and *C. acinaciformis* (1323 μg CH_4_ h^−1^ m^−2^) but the latter two species did not significantly differ from one another (Figure 2b, Table S4).

At the landscape scale, we estimated a combined annual emission of 2 × 10^−9^ Tg CH_4_ ha^−1^ y^−1^ from termite mounds of our three study species (Table S5). *Amitermes laurensis* mounds contributed the most CH_4_ on a per hectare basis (Figure 2c, mean = 1.04 × 10^−9^ Tg CH_4_ ha^−1^ year^−1^), *N. magnus* was the second highest CH_4_ emitter (Figure 2c, mean = 8.17 × 10^−10^ Tg CH_4_ ha^−1^ year^−1^), while *C. acinaciformis* (Figure 2c, mean = 1.69 × 10^−10^ Tg CH_4_ ha^−1^ year^−1^) contributed the least to landscape mound CH_4_ emissions.

### 3.2 Relative Importance of Structural Traits and External Environmental Drivers in Mound‐Level CH_4_ Emission

**FIGURE 3:**
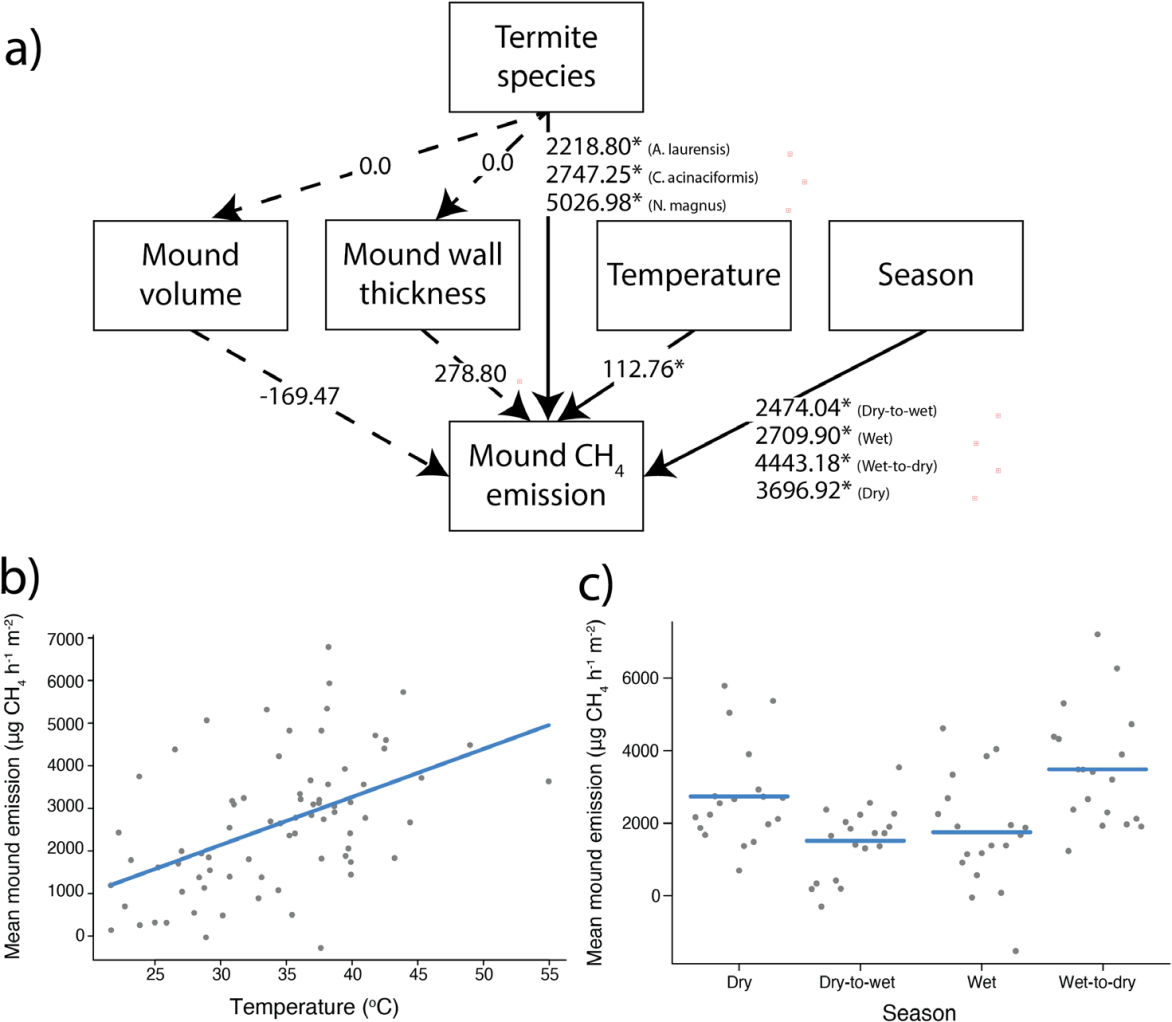
(a) Structural equation modeling diagram showing significant (solid line) and nonsignificant (dashed line) predictors of mound CH_4_ emission. Values on each line indicate *β* values from the SEM, and *β* values for each level of categorical predictors are included. Conditional plots for (b) temperature (°C) and (c) season visualized with *visreg* (Breheny and Burchett 2017). Lines represent model predicted values (for each season) and mean mound CH_4_ emission, while holding all other predictors constant (from Equation 3). Full SEM results can be found in Table S6.

There was a good overall fit to the data (marginal *R* ^2^ = 0.30, conditional *R* ^2^ = 0.68) in the SEM analysis, indicating no significant missing pathways (Fisher's C = 8.372, df = 10, *p* = 0.593). Higher mound surface temperatures were positively associated with increased emissions (*β* = 2808.2, *p* = 0.038, Figure 3, Table S6). Season significantly influenced flux (*p* < 0.001, Figure 3), with CH_4_ emissions peaking during the wet‐to‐dry transition season (*β* = 4443.2, *p* < 0.001, Table S6). Termite species had a marginally significant effect on mean CH_4_ emissions (*p* = 0.064, Figure 3), and mounds constructed by *C. acinaciformis* (*β* = 2747.3, *p* < 0.01, Table S6) and *N. magnus* (*β* = 5027.0, *p* < 0.001, Table S6) exhibited significantly higher CH_4_ emissions than those built by *A. laurensis*.

In contrast, mound volume and mean wall thickness, while hypothesized mediators of mound structure effects on CH_4_ emission, were not significant predictors (volume: *p* = 0.72; wall thickness: *p* = 0.55, Figure 3). Neither volume nor wall thickness differed significantly among species (Table S6).

### 3.3 Between Mound Variation in CH_4_ Emissions: Bacterial Methanotroph Communities and the pmoA Gene

**FIGURE 4:**
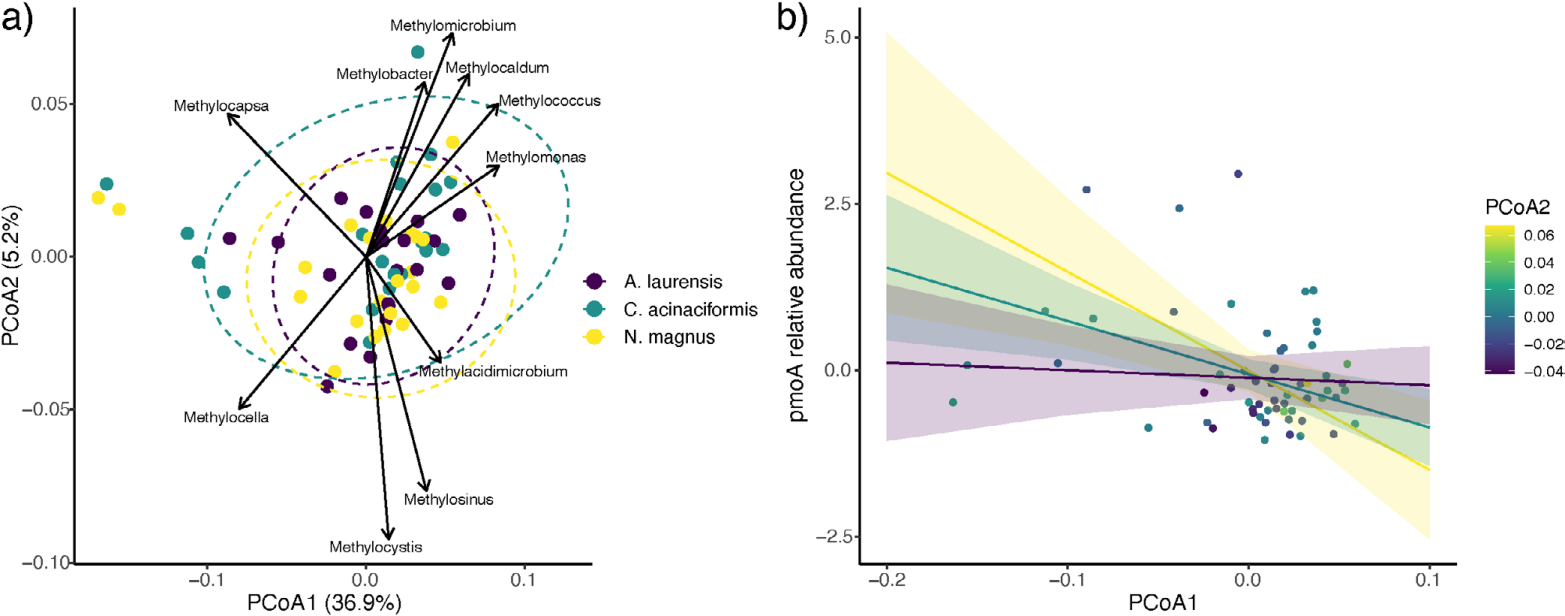
(a) PCoA plot based on Bray–Curtis dissimilarity for genera‐level community composition of methanotrophs in mound material of the three termite study species (denoted by colored points and ellipses). Arrows indicate weighted genera vectors. (b) A significant interaction between PCoA1 and PCoA2 predicting the relative abundance of the *pmoA* gene (yellow line, PCoA2 = 0.06; green line, PCoA2 = 0.1; purple line, PCoA2 = −0.04).

Methanotrophic bacteria represented a small proportion of the bacterial community (0.25% of the total reads) and were dominated by *Methylocystis* sp. (Figure S2). *Methylomicrobium, Methylobacter, Methylocaldum, Methylococcus*, and *Methylomonas* were associated with high PCoA1 and PCoA2 values, which explained 36.9% and 5.2% of variation in the data, respectively. *Methylocapsa* was associated with low PCoA1 and high PCoA2 values, while the opposite was true for *Methylacidimicrobium*, *Methylosinus*, and *Methylocystis* genera. *Methylocella* was the only genus associated with low PCoA1 and PCoA2 values (Figure 4a).

Overall, there were poor links between bacterial methanotrophs and mound CH_4_ emissions. Termite species explained only 1.5% of the variation in methanotroph communities (at the genus level, *R* ^2^ = 0.015, *F* = 0.48, *p* = 0.73) and CH_4_ flux explained only 1.4% (*R* ^2^ = 0.014, *F* = 0.90, *p* = 0.37). There was minimal association between CH_4_ emission and methanotroph community structure in terms of PCoA1 and PCoA2 (Figure 4a).

We found that *pmoA* gene relative abundance was negatively related to PCoA1 (*β* = −7.99, SE = 2.65, df = 55.11, *t* = −3.01, *p* = 0.004, Figure 4b) and not significantly related to PCoA2 scores (*p* = 0.65). However, there was a significant interaction between PCoA1 and PCoA2 (*β* = −343.33, SE = 154.88, df = 56.00, *t* = −2.22, *p* = 0.03, Figure 4b). At high values of PCoA2, there was a negative relationship between PCoA1 and *pmoA* gene relative abundance, while at low values of PCoA2, there was no significant relationship between PCoA1 and *pmoA* gene relative abundance.

Finally, CH_4_ emissions were not significantly predicted by relative abundance of *pmoA*, the sum of all methanotroph relative abundances, termite species, or PCoA1 and PCoA2 scores and their interaction (Table S7). Additionally, the relative abundance of any single methanotroph bacterial genus was not significantly related to CH_4_ emission (Table S8).

### 3.4 Within Mound Variation in Methane Emissions: Bacterial Methanotroph Communities and the pmoA Gene

For those mounds with high intra‐mound variation in CH_4_ emissions, *N. magnus* mounds emitted marginally more CH_4_ than *A. laurensis* mounds (*β* = 3420.91, SE = 1533.97, df = 11.32, *t* = 2.23, *p* = 0.047), but no other predictors (relative abundance of *pmoA*, sum of all methanotroph relative abundances, PCoA1 and PCoA2 and their interaction) were significant (Table S9).

## 4 Discussion

In this study we sought to understand how the internal and external termite mound environment shaped CH_4_ emission by measuring the influence of species‐level differences for various spatial scales, mound structural traits, seasons, temperatures, and methanotroph communities. We showed that the termite species contributing the most to CH_4_ emissions differed at the individual, mound, and landscape‐scale. Differences at the landscape‐scale are likely due to mound abundance, and while not directly tested, differences at the mound‐level could be driven by differences in termite biomass. Given that most CH_4_ is likely emitted by termites inside mounds, accounting for the internal mound environment should be a critical consideration in calculating net CH_4_ emission. Factors controlling termite mound emissions are therefore an important consideration for accurately predicting termite contributions to the CH_4_ budget. From our work, mound CH_4_ emissions were most strongly influenced by termite species inhabiting the mounds, temperature, and season. Furthermore, while microbial methanotrophy within the mound has been shown to be an important filter controlling net CH_4_ release, we did not find direct associations between either the bacterial methanotroph communities or the *pmoA* gene abundances and CH_4_ release. However, the relationship between methanotroph communities and the *pmoA* gene suggests that expression, rather than relative abundance, could reveal the interplay between termite methanogenesis, bacterial methanotrophy, and net mound CH_4_ emission. Below we further explore the findings of this field study and discuss how incorporating scale, species‐level differences, external environmental conditions, and methanotrophic microbes into CH_4_ modeling can contribute to accurately estimating termites' role in global CH_4_ budgets.

### 4.1 Species Contributions to CH_4_ Emissions Differ Depending on the Scale of Measurement

The scale of measurement mattered for determining the relative role of different termite species in contributing to CH_4_ emission. *Coptotermes acinaciformis* individuals had the highest TEF values, *N. magnus* had the highest mound‐level emissions, and when landscape‐level mound density was factored into CH_4_ emission calculations, *A. laurensis* mounds released the most CH_4_. At the individual scale, variation between species was most likely underpinned by differences in termite diet and the gut microbial community that each species harbors (Clement 2022). While we were not able to directly test the effect of diet on CH_4_ emission, there are known differences in how CH_4_ varies by feeding group (Sanderson 1996; Zhou et al. 2022), which is ultimately controlled by termite gut microbial composition, specifically the presence and abundance of methanogenic microbes (Rouland et al. 1993). Interestingly, *Coptotermes acinaciformis*, which had the highest TEF in our study (contrasting Sanderson 1996 findings on wood‐feeders), has been shown to have a greater abundance of an archaeal methanogenic gut microbe (*Methanobrevibacter* sp.) compared to *N. magnus* and *A. laurensis* (from data collected at the same study site, Clement 2022). While we observed high CH_4_ production for *C. acinaciformis* individuals, consistent with a greater abundance of methanogenic gut microbes (Clement 2022), we found no evidence of microbial methanotrophy reducing CH_4_ emissions at the mound‐level. To explain such discrepancies between individual and mound‐level emissions, further investigation of the balance of methanogenesis within termites and methanotrophy occurring in the termite mound is necessary.

Differences in top emitting species at the mound and landscape scale were underpinned by tradeoffs between high average mound emissions and high termite mound densities. *Nasutitermes magnus* mounds were larger (mean surface area = 4.5 m^2^) and had an average mound emission (3426 μg CH_4_ h^−1^ m^−2^) that was greater than *A. laurensis* (1270 μg CH_4_ h^−1^ m^−2^). Greater emissions from *N. magnus* mounds could result from more termite biomass housed in larger mounds, although mound size is an imperfect indicator of colony size (Josens and Soki 2010). However, *A. laurensis* mounds, although they were smaller (mean surface area = 0.7 m^2^) were approximately 22 times more abundant compared to *N. magnus* mounds per hectare, leading to a greater overall contribution by *A. laurenesis* when CH_4_ was estimated annually per hectare (1.04 × 10^−9^ > 8.17 × 10^−10^ Tg CH_4_ ha^−1^ year^−1^). We propose that in tropical savannas and other ecosystems with high mound abundance, combining entire mound CH_4_ measurements with landscape‐scale mound density to calculate a “mound emission factor” per area can better represent ecosystem CH_4_ emission, rather than scaling up with a TEF which is common practice (Saunois et al. 2020; Ito 2023).

### 4.2 Temperature and Season Impact CH_4_ Emission More Than Mound Structure

The differences in CH_4_ emissions that we found at various scales of measurement (individual, mound, landscape) also emphasize the importance of the “colony context”. Methane emissions from termites are largely released from workers inside the mound. Thus, net CH_4_ emissions are not only shaped by production from gut methanogens within termites (Brune and Dietrich 2015), but also by mound structure, microbial methanotrophy, and the external environmental conditions (i.e., temperature and seasonality, Korb 2003; Singh et al. 2019; Nauer, Hutley, and Arndt 2018; Jamali, Livesley, Dawes, Hutley, and Arndt 2011). In our ecosystem, temperature and season emerged as strong predictors of CH_4_ emission, contributing to the growing body of literature suggesting termites and termite‐driven decomposition are highly sensitive to external environmental conditions (Zanne et al. 2022; Law, Flores‐Moreno, et al. 2024).

Interestingly, we found that CH_4_ emissions in the wet‐to‐dry transition season were significantly greater than the mid‐wet and dry‐to‐wet seasons. Our results revealed far less seasonal variation in mound CH_4_ emission compared to previous studies, as wet‐to‐dry transition season emissions were only 28%–33% higher than the dry‐to‐wet and mid‐wet seasons. In contrast, one study in northern Australia reported up to 26‐fold higher emissions in the wet compared to the dry season for *Microcerotermes nervosus* mounds (Jamali, Livesley, Dawes, Cook, et al. 2011), and another study in Africa showed that wet season emissions were 64% lower than the dry season (Räsänen et al. 2023). Our wet season CH_4_ measurements followed Cyclone Jasper, which flooded parts of the site; it is possible that this anomalous inundation altered seasonal termite population dynamics and concealed some of the extreme wet season dynamics in CH_4_ emission observed by Jamali, Livesley, Dawes, Hutley, and Arndt (2011). This study and others showed that seasonality can influence CH_4_ emission, but across the studies, there is not strong consensus on which season has the greatest emissions. These differences merit a closer investigation of the underlying factors driving seasonal shifts. Field studies investigating fluctuations in the termite mound population with alterations to water and food resources (Jamali, Livesley, Dawes, Hutley, and Arndt 2011), as well as interactions between temperature and moisture availability on the metabolic functioning of both termites and microbes, are suggested avenues of future research.

As temperatures increased so too did mound CH_4_ emission, pointing to climatic regulation of termite metabolic activity (Jamali, Livesley, Dawes, Hutley, and Arndt 2011; Zanne et al. 2022), as well as the methanogenic microbes in termite guts. The relationship that we define between temperature and CH_4_ emissions can help to parameterize biogeochemical cycling models that could be used to understand how termite mound CH_4_ emissions, at savanna and global scales, shift with future climates (Zanne et al. 2022). Our results are especially relevant as studies project that termite‐mediated decomposition rates respond positively to increased temperatures (Zanne et al. 2022), and termite range expansion is another predicted consequence of climate warming (Zanne et al. 2022; Ito 2023). Ito (2023) estimated that from elevated CO_2_ (via vegetation productivity), climatic warming, and land‐use change, termite CH_4_ emissions could increase between 0.5–5.9 Tg year^−1^. Given that our study provides species‐specific, field‐based measurements to define a temperature‐emission relationship, this could serve as a foundation to evaluate the validity of such claims.

Additionally, when predicting if emissions will increase due to higher temperatures it is necessary to consider which termite species will expand in range and what nesting behaviors they have (Law, Allison, et al. 2024). Increases in subterranean‐dwelling termites are unlikely to affect net CH_4_ emission (Eggleton et al. 1999), but mound‐building species are better adapted to temperature change as the mound structure can maintain internal homeostasis (Korb 2011; Wijas et al. 2022). As such, significant additional CH_4_ emissions from mound building termites could result from the combination of warmer ambient temperatures and range expansion. Modifying existing ecosystem models to include parameters for termites, including termite CH_4_ generation, range expansion, and microbial oxidation occurring within the mound would be a step forward in accounting for such shifts (Law, Allison, et al. 2024). For one northern Australian termite species (*Microcerotermes* sp.), Law, Allison, et al. (2024) showed that CH_4_ production decreased above 35°C, suggesting that there may be an upper limit CH_4_ emission, yet our models showed that CH_4_ emission continued to increase above this temperature. It is therefore important to quantify how termites and termite‐associated microbial functions respond more broadly to temperature increases and if a thermal maximum limits CH_4_ emissions at the mound level. Given that we measured three species of termites, we provide a small insight as to how termite CH_4_ emissions can respond to changing temperatures. Notably these are the dominant species at our site and are therefore the main contributors to carbon dynamics in our study ecosystem. Moving forward, relationships between termite mound CH_4_ emission and temperature should be tested in other ecosystems and across a range of species, which would be useful for informing parameterization of the global CH_4_ budget.

### 4.3 The Influence of Mound Methanotroph Communities on Functional Genes, but Not CH_4_ Emission

In our analysis of metagenomic data from termite mound material we found that specific combinations of the bacterial methanotroph community (represented by PCoA1 and PCoA2 scores) jointly determined the response of CH_4_ emission. Interestingly, methanotrophs were rare members of the mound bacterial community (0.25% of all genera), which is in accordance with results reported by Chiri et al. (2020). Of the 16 methanotrophic bacterial genera that we identified, 10 genera were found in the termite mound material we sampled, suggesting that termite mounds harbor a diverse methanotroph community. While we showed no species‐level differences in methanotrophic members in mound material, future work to characterize methanotrophs found in the mounds of other species in other places would help to determine if the methanotroph communities assembling in termite mounds are broadly consistent.

Surprisingly, we were unable to directly link local methanotroph communities or *pmoA* gene abundance to lower CH_4_ emissions. In contrast, Nauer, Hutley, and Arndt (2018) showed that 50% of termite‐produced CH_4_ was oxidized on average prior to leaving the mound for three North Australian termite species (*Microcerotermes nervosus*, *Macrognathotermes sunteri*, and *Tumulitermes pastinator*), suggesting that mound methanotrophs play an important role in net CH_4_ release. It appears that the missing link between methanotrophy potential, as indicated by relative abundances, and the result of CH_4_ oxidation in termite mounds is an understanding of *pmoA* expression. Metatranscriptomics could be used to better identify the direct function of bacterial methanotrophy and its subsequent impacts on CH_4_ emission. Future studies should pursue a more integrated approach with field and laboratory based CH_4_ measurements and microbial ecology, using both metagenomics and metatranscriptomics to determine environmental and community‐based methanotrophic controls on net CH_4_ emission from termite mounds. For example, the controlled environment of mound mesocosm experiments could be used to study how mound methanotrophs are activated over time, by season or with temperature, which could subsequently be related to CH_4_ emissions from mounds and individual termites (Law, Allison, et al. 2024). Furthermore, there is significant opportunity to holistically describe the termite mound microbiome, yet such sampling may be difficult to pair with emission measurements in the field, as it is highly destructive (Chiri et al. 2020). Lastly, mapping methanotroph‐associated gene expression across a range of external environmental conditions in the field, particularly temperature, would provide insight as to the temporal and climate‐mediated shifts in methanotrophy within the mound.

### 4.4 What Is the Most Effective Way to Upscale CH_4_ Emissions From Termite Mounds?

To complement the TEF and biomass approach, mound‐level CH_4_ emission factors may have greater utility when applied in mound‐dominated landscapes to quantify CH_4_ emissions. Emissions at the mound‐level have been recorded for a growing number of species across tropical ecosystems (Table S10), and while not as robustly characterized as TEFs (Zhou et al. 2022), collecting CH_4_ emission data across the diversity of mound‐building termites is a key research objective for improving global estimates (Law, Allison, et al. 2024). It will be important for future studies to more accurately map termite biomass by nesting strategy at a global scale, so that the mound‐level emission factor approach can be applied where it is best suited. More generally, we challenge researchers to consider how the environment surrounding termites may alter the amount of CH_4_ that reaches the atmosphere. Additionally, given the variability in termite mound sizes, it is useful to pair mound‐level emission factors with average mound sizes. Photogrammetry is an accessible tool for this, as it is a portable method utilizing a simple smartphone app and a small amount of post‐processing that can be done in open‐source programs (i.e., Polycam, MeshLab, Blender).

Even with a broad database of mound‐level emission factors, there remains the challenge of determining landscape‐level mound abundance (Law, Allison, et al. 2024). Termite mounds range in spatial distribution, demonstrating both aggregated and more evenly distributed patterns (Davies et al. 2014; Levick et al. 2010), making field surveys challenging. Technologies such as airborne Lidar allow for epigeal mounds to be detected and mapped over large areas with greater ease (Levick et al. 2010; D'hont et al. 2021) and could be used to quantify mound dimensions (i.e., height and volume, D'hont et al. 2021). However, determining if remotely‐sensed mounds are active and occupied by termites remains an important limitation, but can be resolved with field data ground truthing the proportion of mounds active in an ecosystem (Clement et al. 2021). Paired with broader characterization of mound‐level CH_4_ emission across the termite diversity of a given region, remotely sensed termite mound abundance data will substantially reduce uncertainty in CH_4_ accounting efforts.

## 5 Conclusion

Our findings highlight both the complexity and the opportunity in refining global termite CH_4_ emissions by incorporating the context and dynamics of the internal and external termite mound environment. Termite species, temperature, and season emerge as important factors that should be used to improve estimates of global termite CH_4_ emissions. Future research directly linking methanotrophy‐associated gene expression in microbes within mounds to CH_4_ oxidation could help define a microbial oxidation factor applicable to mound CH_4_ emissions on a larger scale. Additionally, measuring mound‐level emission factors for the global diversity of mound‐building species can incorporate microbial oxidation and relieve errors inherent in calculations relying only on TEFs and termite biomass. Our understanding of the factors influencing termite mound methane in Australian savannas can be used to refine termite contributions to the global CH_4_ budget.

## Author Contributions

**Abbey R. Yatsko:** conceptualization, methodology, investigation, writing – original draft, formal analysis. **Caleb Jones:** investigation, writing – review and editing. **Paul Eggleton:** formal analysis, writing – review and editing. **Baptiste Wijas:** investigation, formal analysis, writing – review and editing. **Amy E. Zanne:** conceptualization, writing – review and editing, supervision. **Jeff R. Powell:** formal analysis, writing – review and editing. **Marcos Pérez‐Losada:** formal analysis. **Ignacio Ramos‐Tapia:** formal analysis.

## Funding

This work was supported by the University of Miami Biology Department and a National Science Foundation Graduate Research Fellowship (Award #1938060) to ARY. Funding was also acquired from the US National Science Foundation, Ecosystem Studies Cluster, under awards DEB‐1655759 and DEB‐2149151 to AEZ as well as a UK NERC grant NE/K01613X/1 to PE.

## Conflicts of Interest

The authors declare no conflicts of interest.

## Supporting information


**Figure S1:** Diagram of chamber‐based sampling for larger mounds (five measurement points, for *C. acinaciformis* and *N. magnus* ). (a) A *N. magnus* mound in the field with sampling chambers fixed in place. (b) The semi‐closed sampling chamber system uses an external ring of potting clay to maintain airtight conditions. Tubing coming out from the chamber connects to the LGR UGGA. (c) Schematic for sampling larger mounds at five locations on each mound: north‐facing (N), east‐facing (E), south‐facing (S), west‐facing (W), and top (T). Note that in the case of *A. laurensis* mounds, the top (T) measurement was omitted.
**Figure S2:** Relative abundance of the ten methanotroph genera identified in the mound material metagenomic dataset for evaluating variation between mounds.
**Figure S3:** Example of how data was recorded during the measurement of individual termite CH_4_ emissions (expressed as ppm CH_4_). Red indicates where the measurement chamber is inactive and open, therefore only recording concentration of CH_4_ in ambient air. Yellow indicates the calibration period, where the chamber is closed but measurements are not yet recorded. Green indicates the measurement period, where the chamber is closed and measurements are being made of changes to CH_4_ concentration. This measurement came from 2.17 g of *N. magnus* individuals, which had a TEF of 0.33 μg CH4 h^−1^ g termite^−1^, which was calculated from the slope of the line in the measurement period.
**Table S1:** Environmental conditions and mound CH_4_ emission sample sizes for the four resampling campaigns based on season. Monthly average temperatures for May 2022, November 2022, and August 2023 were derived from a weather station at the field site. The average temperature in February 2024 was sourced from the NASA POWER dataset using the R package “nasapower” (Sparks 2018) due to the fact that in December 2023 Cyclone Jasper destroyed the field site weather station. Precipitation data for each season is calculated as the 1 month average precipitation prior to field sampling and was sourced from the Australian Bureau of Meteorology (BOM 2024).
**Table S2:** Description of mounds re‐measured across the four sampling campaigns. Bolded mound IDs indicate mounds that were successfully measured in each of the four campaigns. If a mound was destroyed or died, it is indicated when this occurred and what mound served as the replacement.
**Table S3:** Summary statistics for post hoc pairwise comparisons to determine significant species‐level differences in TEF values. Bolded *p*‐values indicate significant pairwise contrasts.
**Table S4:** Summary statistics for post hoc pairwise comparisons to determine significant differences between mound‐level CH_4_ emissions for the three study species. Bolded *p*‐values indicate significant pairwise contrasts.
**Table S5:** Results summarized across all levels of CH_4_ emission: termite individual (TEF), average mound‐level (per mound), and landscape scale (per hectare). Data for the mounds occupied per ha come from Clement et al. (2021). Average TEF units: μg CH_4_ g^−1^ termite h^−1^. Average mound emission units: Tg CH_4_ mound^−1^ year^−1^. Landscape‐scale mound emission units: Tg CH_4_ ha^−1^ year^−1^. Asterisks (*) in the average TEF, average mound‐level emission, and landscape‐scale mound emission columns indicate the top‐emitting species at each level of inference (individual, mound, landscape).
**Table S6:** Summary of structural equation modeling (SEM) output.
**Table S7:** Model summary of linear mixed effects model testing all relevant predictors of CH_4_ emission to understand variation across mounds. For termite species‐level comparisons, *A. laurensis* is the reference species.
**Table S8:** Model results for testing individual methanotroph genera relative abundance as a predictor of CH_4_ emission. Bolded values indicate significance.
**Table S9:** Model summary of linear mixed effects model testing all relevant predictors of CH_4_ emission to understand variation within mounds. For termite species‐level comparisons, *A. laurensis* is the reference species.
**Table S10:** Summary of published CH_4_ emissions for termite mounds at the species level. Note that the sample sizes from this study include mounds that were a part of the initial, larger sampling campaign, hence the greater sample size for each species.
**Table S11:** List of bacterial genera associated with methanotrophy. These genera were used to query the metagenomics dataset for methanotroph candidates to include in our analysis on variation in bacterial methanotroph community composition within and between mounds.
**Table S12:** Coefficient of Variation (CV) in CH_4_ emissions for individual mounds (calculated from 4 to 5 emission subsamples) which were remeasured in all four seasons.
**Table S13:** Mean mound size (as surface area, m^2^ and as volume, m^3^) and mound wall thickness (mm) for the three study species.

## Data Availability

Data and code have been uploaded to Zenodo at https://doi.org/10.5281/zenodo.19141664 (Yatsko 2026). The metagenomic data supporting this study are available under Bioproject PRJNA1234298 at https://www.ncbi.nlm.nih.gov/bioproject/PRJNA1234298.
