# Supplementary material for: Rethinking Termite Methane Emissions: Does the Mound Environment Matter?
Source: Glob Chang Biol. 2026 Apr 9;32(4):e70838. doi: 10.1111/gcb.70838 (PMC13063211; doi:10.1111/gcb.70838)
Supplement: Supplementary file 1 — Figure S1: Diagram of chamber‐based sampling for larger mounds (five measurement points, for C. acinaciformis and N. magnus ). (a) A N. magnus mound in the field with sampling chambers fixed in place. (b) The semi‐closed sampling chamber system uses an external ring of potting clay to maintain airtight conditions. Tubing coming out from the chamber connects to the LGR UGGA. (c) Schematic for sampling larger mounds at five locations on each mound: north‐facing (N), east‐facing (E), south‐facing (S), west‐facing (W), and top (T). Note that in the case of A. laurensis mounds, the top (T) measurement was omitted. Figure S2: Relative abundance of the ten methanotroph genera identified in the mound material metagenomic dataset for evaluating variation between mounds. Figure S3: Example of how data was recorded during the measurement of individual termite CH4 emissions (expressed as ppm CH4). Red indicates where the measurement chamber is inactive and open, therefore only recording concentration of CH4 in ambient air. Yellow indicates the calibration period, where the chamber is closed but measurements are not yet recorded. Green indicates the measurement period, where the chamber is closed and measurements are being made of changes to CH4 concentration. This measurement came from 2.17 g of N. magnus individuals, which had a TEF of 0.33 μg CH4 h−1 g termite−1, which was calculated from the slope of the line in the measurement period. Table S1: Environmental conditions and mound CH4 emission sample sizes for the four resampling campaigns based on season. Monthly average temperatures for May 2022, November 2022, and August 2023 were derived from a weather station at the field site. The average temperature in February 2024 was sourced from the NASA POWER dataset using the R package “nasapower” (Sparks 2018) due to the fact that in December 2023 Cyclone Jasper destroyed the field site weather station. Precipitation data for each season is calculated as the 1 month average prec [file GCB-32-e70838-s001.docx]

**Supplementary Information**


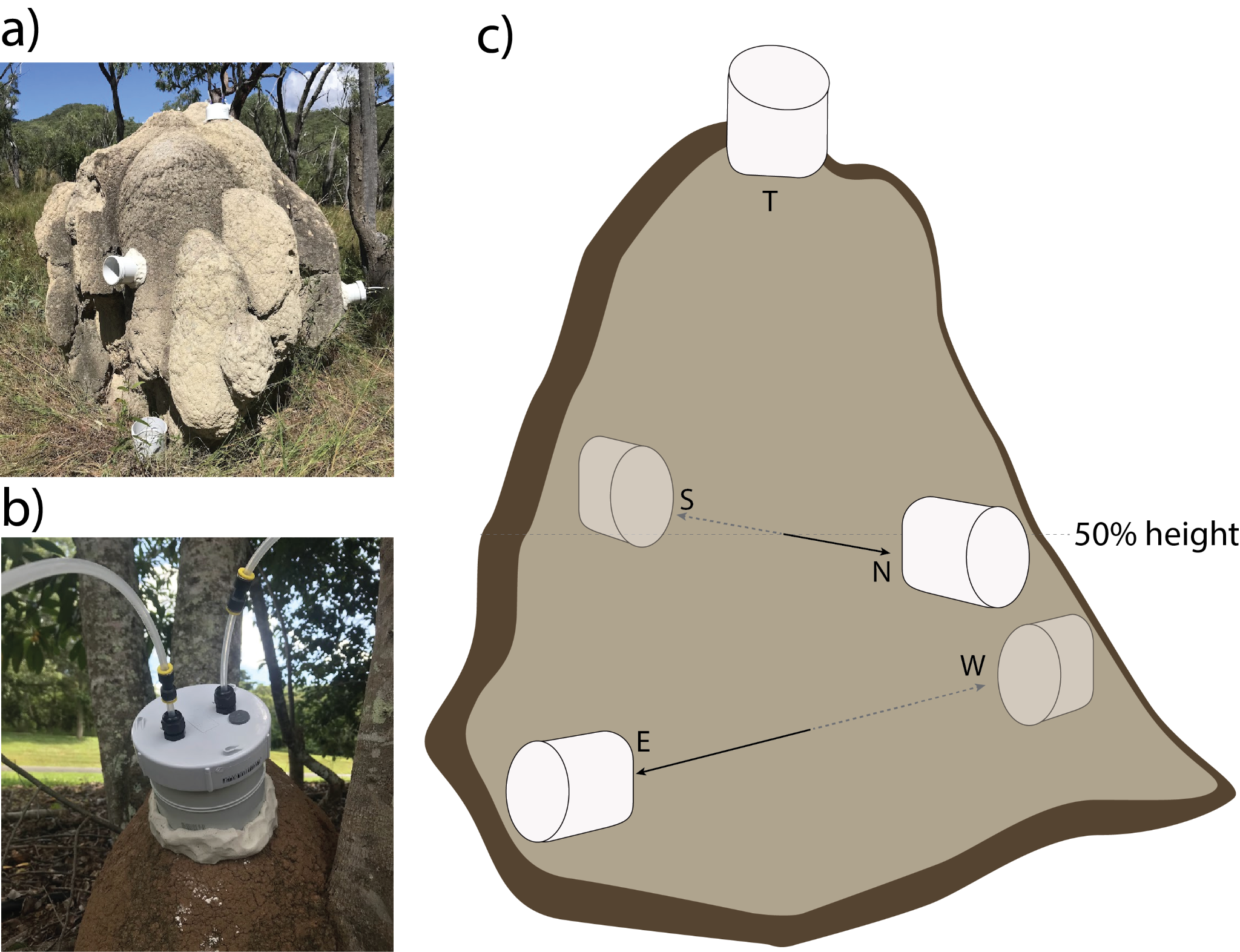


Supplementary Figure 1. Diagram of chamber-based sampling for larger mounds (five measurement points, for *C. acinaciformis* and *N. magnus*). a) A *N. magnus* mound in the field with sampling chambers fixed in place. b) The semi-closed sampling chamber system uses an external ring of potting clay to maintain airtight conditions. Tubing coming out from the chamber connects to the LGR UGGA. c) Schematic for sampling larger mounds at five locations on each mound: north-facing (N), east-facing (E), south-facing (S), west-facing (W), and top (T). Note that in the case of *A. laurensis* mounds, the top (T) measurement was omitted.


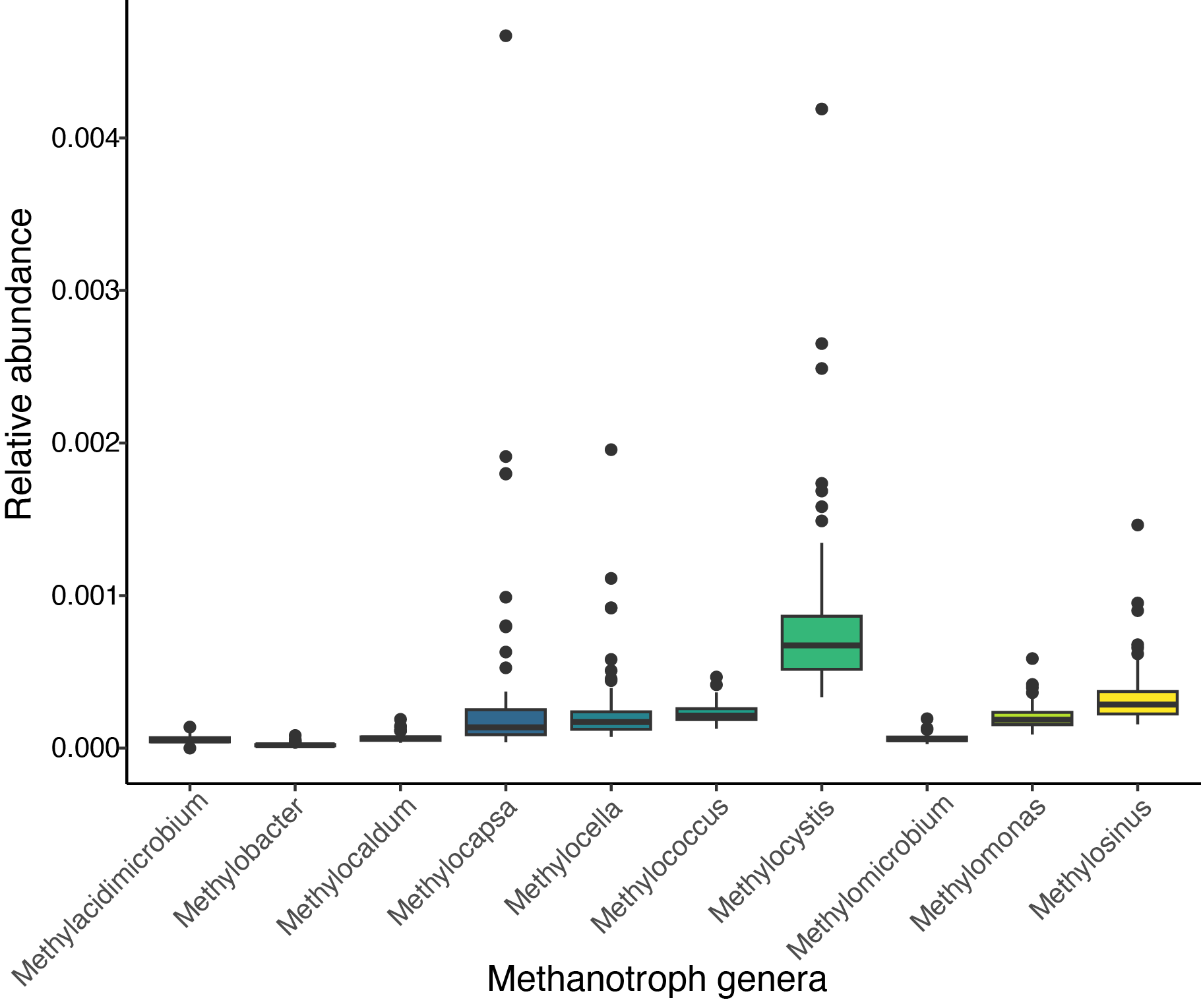


Supplementary Figure 2. Relative abundance of the ten methanotroph genera identified in the mound material metagenomic dataset for evaluating variation between mounds.


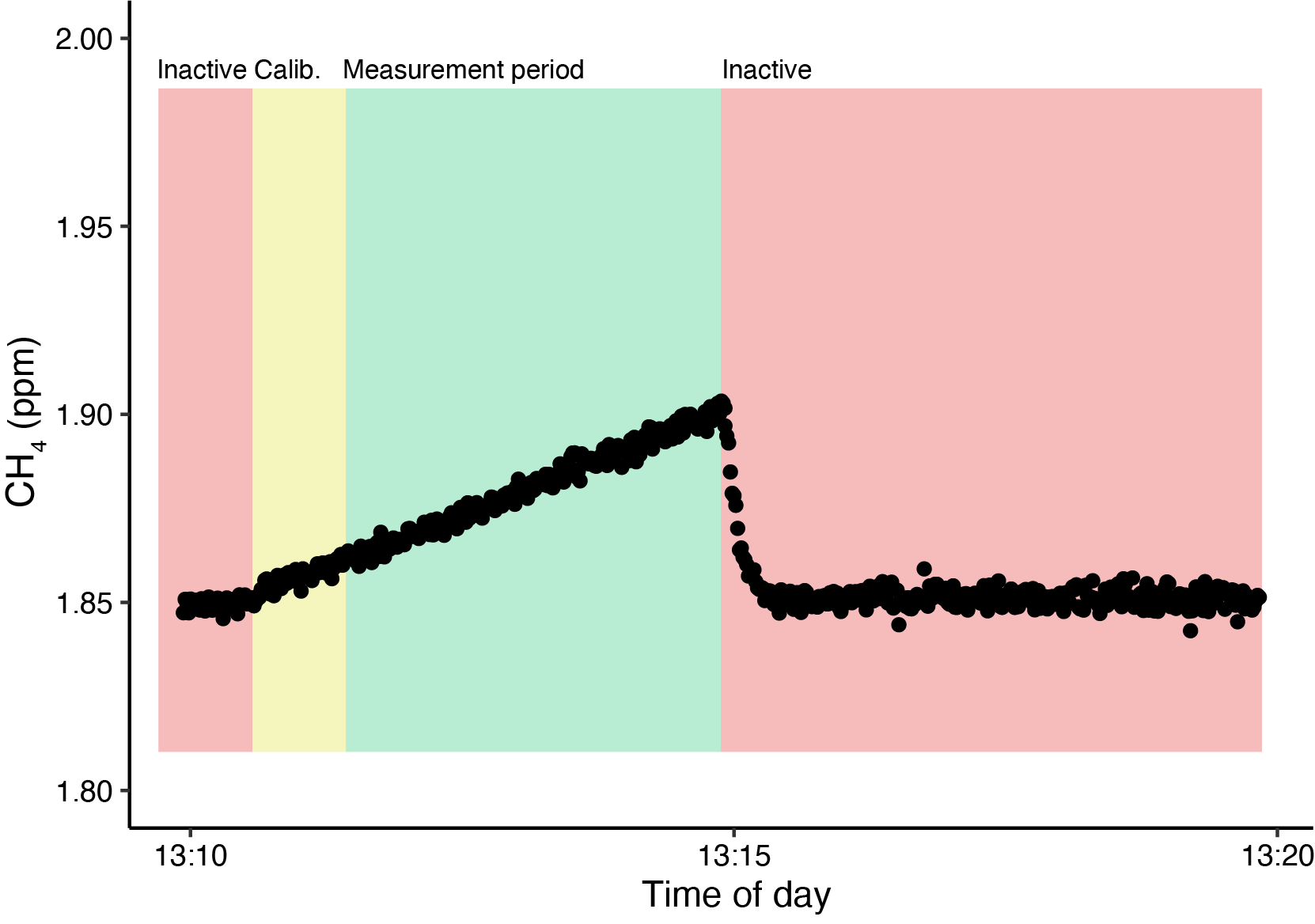


Supplementary Figure 3. Example of how data was recorded during the measurement of individual termite CH_4_ emissions (expressed as ppm CH_4_). Red indicates where the measurement chamber is inactive and open, therefore only recording concentration of CH_4_ in ambient air. Yellow indicates the calibration period, where the chamber is closed but measurements are not yet recorded. Green indicates the measurement period, where the chamber is closed and measurements are being made of changes to CH_4_ concentration. This measurement came from 2.17 g of *N. magnus* individuals, which had a TEF of 0.33 μg CH4 h^-1^ g termite^-1^, which was calculated from the slope of the line in the measurement period.

Supplementary Table 1. Environmental conditions and mound CH_4_ emission sample sizes for the four resampling campaigns based on season. Monthly average temperatures for May 2022, November 2022, and August 2023 were derived from a weather station at the field site. The average temperature in February 2024 was sourced from the NASA POWER dataset using the R package ‘nasapower’ (Sparks 2018) due to the fact that in December 2023 Cyclone Jasper destroyed the field site weather station. Precipitation data for each season is calculated as the 1 month average precipitation prior to field sampling and was sourced from the Australian Bureau of Meteorology (2024).

| Sampling campaign | | Environmental conditions | | Total mounds measured | | |
| --- | --- | --- | --- | --- | --- | --- |
| Date | Season | Temperature  (C) | Precipitation  (mm, 1 month average) | *A. laurensis* (n) | *C. acinaciformis* (n) | *N. magnus* (n) |
| May 2022 | Wet-to-dry | 22.4 | 129.9 | 14 | 25 | 21 |
| Nov 2022 | Dry-to-wet | 26.3 | 28.0 | 8 | 8 | 8 |
| Aug 2023 | Dry | 19.2 | 17.1 | 8 | 8 | 8 |
| Feb 2024 | Wet | 25.1 | 160.2 | 8 | 8 | 7 |

Supplementary Table 2. Description of mounds re-measured across the four sampling campaigns. Bolded mound IDs indicate mounds that were successfully measured in each of the four campaigns. If a mound was destroyed or died, it is indicated when this occurred and what mound served as the replacement.

| Species | Mound ID | May 2022  measurement | November 2022  measurement | August 2023  measurement | February 2024  measurement |
| --- | --- | --- | --- | --- | --- |
| *A. laurensis* | **MD10** | X | X | X | X |
|  | **MD15** | X | X | X | X |
|  | MD21 | X | X | Removed, replaced by MD22 | n/a |
|  | **MD24** | X | X | X | X |
|  | **MD36** | X | X | X | X |
|  | **MD37** | X | X | X | X |
|  | **MD42** | X | X | X | X |
|  | **MD54** | X | X | X | X |
|  | MD22 | X | n/a | X | X |
| *C. acinaciformis* | **MD2** | X | X | X | X |
|  | **MD26** | X | X | X | X |
|  | **MD27** | X | X | X | X |
|  | MD35 | X | X | X | Removed, replaced by MD9 |
|  | **MD48** | X | X | X | X |
|  | **MD55** | X | X | X | X |
|  | **MD59** | X | X | X | X |
|  | **MD63** | X | X | X | X |
|  | MD9 | X | n/a | n/a | X |
| *N. magnus* | **MD11** | X | X | X | X |
|  | **MD25** | X | X | X | X |
|  | **MD50** | X | X | X | X |
|  | MD52 | X | X | X | Removed, not replaced |
|  | MD56 | X | X | Removed, replaced by MD32 | n/a |
|  | MD57 | X | X | Removed, replaced by MD61 | n/a |
|  | **MD58** | X | X | X | X |
|  | **MD65** | X | X | X | X |
|  | MD32 | X | n/a | X | X |
|  | MD61 | X | n/a | X | X |

Supplementary Table 3. Summary statistics for post hoc pairwise comparisons to determine significant species-level differences in TEF values. Bolded p-values indicate significant pairwise contrasts.

| Species contrast | estimate | SE | df | t ratio | p-value |
| --- | --- | --- | --- | --- | --- |
| *A. laurensis - C. acinaciformis* | -0.343 | 0.104 | 12 | -3.291 | **0.02*** |
| *A. laurensis - N. magnus* | 0.360 | 0.104 | 12 | 3.450 | **0.01*** |
| *C. acinaciformis - N. magnus* | 0.703 | 0.104 | 12 | 6.741 | **0.0001*** |

Supplementary Table 4. Summary statistics for post hoc pairwise comparisons to determine significant differences between mound-level CH_4_ emissions for the three study species. Bolded p-values indicate significant pairwise contrasts.

| Species contrast | estimate | SE | df | t ratio | p-value |
| --- | --- | --- | --- | --- | --- |
| *A. laurensis - C. acinaciformis* | -107 | 534 | 134 | -0.201 | 0.98 |
| *A. laurensis - N. magnus* | 0.360 | 549 | 135 | -5.786 | **< 0.0001*** |
| *C. acinaciformis - N. magnus* | 0.703 | 501 | 121 | -6.126 | **< 0.0001*** |

Supplementary Table 5. Results summarised across all levels of CH_4_ emission: termite individual (TEF), average mound-level (per mound), and landscape scale (per hectare). Data for the mounds occupied per ha come from Clement et al. (2021). Average TEF units: μg CH_4_ g^-1^ termite h^-1^. Average mound emission units: Tg CH_4_ mound^-1^ yr^-1^. Landscape-scale mound emission units: Tg CH_4_ ha^-1^ yr^-1^. Asterisks (*) in the average TEF, average mound-level emission, and landscape-scale mound emission columns indicate the top-emitting species at each level of inference (individual, mound, landscape).

| Species | Feeding group | Average TEF | Mounds occupied ha^-1^ | Average mound-level emission | SD mound-level emission | Landscape-scale mound emissions |
| --- | --- | --- | --- | --- | --- | --- |
| *A. laurensis* | plant leaf litter | 0.73 | 120.4 | 8.64 × 10^-12^ | 1.01 × 10^-11^ | 1.04 × 10^-9^* |
| *C. acinaciformis* | wood | 1.07***** | 2.8 | 6.04 × 10^-11^ | 4.64 × 10^-11^ | 1.69 × 10^-10^ |
| *N. magnus* | grass | 0.37 | 5.6 | 1.46 × 10^-10^* | 1.03 × 10^-10^ | 8.17 × 10^-10^ |
| **All species** | **–** | **0.72** | **128.8** | **7.16 × 10^-11^** | **5.32 × 10^-11^** | **2.03 × 10^-9^** |

Supplementary Table 6. Summary of structural equation modeling (SEM) output.

| Response | Predictor | Estimate | Std. Error | DF | t-value | P-value |
| --- | --- | --- | --- | --- | --- | --- |
| Mound volume | species_s | – | – | 2 | 0.00 | 1.00 |
|  | species_s = N. magnus | 0.00 | 0.22 | 73 | 0.00 | 1.00 |
|  | species_s = C. acinaciformis | 0.00 | 0.19 | 73 | 0.00 | 1.00 |
|  | species_s = A. laurensis | 0.00 | 0.19 | 73 | 0.00 | 1.00 |
| Mound wall thickness | species_s | – | – | 2 | 0.00 | 1.00 |
|  | species_s = A. laurensis | 0.00 | 0.19 | 73 | 0.00 | 1.00 |
|  | species_s = C. acinaciformis | 0.00 | 0.19 | 73 | 0.00 | 1.00 |
|  | species_s = N. magnus | 0.00 | 0.22 | 73 | 0.00 | 1.00 |
| Mean mound CH_4_ emission | volume_m3_st | -169.47 | 458.58 | 14.34 | -0.37 | 0.71 |
|  | wt_mm_st | 278.80 | 455.62 | 14.00 | 0.61 | 0.55 |
|  | temp | 112.76 | 52.58 | 59.23 | 2.14 | **0.04*** |
|  | species_s | – | – | 2 | 3.36 | 0.06 |
|  | species_s = A. laurensis | 2218.80 | 720.44 | 13.97 | 3.08 | **0.008*** |
|  | species_s = C. acinaciformis | 2747.25 | 719.77 | 13.93 | 3.82 | **0.002*** |
|  | species_s = N. magnus | 5026.98 | 856.97 | 14.25 | 5.87 | **<0.0001*** |
|  | campaign | – | – | 3 | 4.52 | **0.007*** |
|  | campaign = nov22 | 2474.04 | 645.34 | 45.16 | 3.83 | **0.0004*** |
|  | campaign = feb24 | 2709.90 | 557.92 | 31.98 | 4.86 | **<0.0001*** |
|  | campaign = aug23 | 3696.92 | 684.67 | 50.19 | 5.40 | **<0.0001*** |
|  | campaign = may22 | 4443.18 | 542.64 | 29.56 | 8.19 | **<0.0001*** |

Supplementary Table 7. Model summary of linear mixed effects model testing all relevant predictors of CH_4_ emission to understand variation across mounds. For termite species-level comparisons, *A. laurensis* is the reference species.

| Fixed effects | Estimate | Std. Error | df | t value | p |
| --- | --- | --- | --- | --- | --- |
| intercept | 3054.89 | 1539.61 | 34.74 | 1.98 | 0.06 |
| pmoA relative abundance | -274.52 | 422.67 | 39.53 | -0.65 | 0.52 |
| Species - *C. acinaciformis* | -821.89 | 1775.22 | 18.48 | -0.46 | 0.65 |
| Species - *N. magnus* | 1222.07 | 1764.64 | 18.10 | 0.70 | 0.50 |
| PCoA1 | 16524.09 | 15631.67 | 38.13 | 1.06 | 0.30 |
| PCoA2 | 2939.86 | 221396.66 | 45.78 | 0.14 | 0.89 |
| Relative abundance methanotroph sum | 284927.70 | 384117.34 | 38.62 | 0.74 | 0.46 |
| PCoA1 * PCoA2 | -392290.96 | 526711.81 | 39.67 | -0.75 | 0.46 |

Supplementary Table 8. Model results for testing individual methanotroph genera relative abundance as a predictor of CH_4_ emission. Bolded values indicate significance.

| Methanotroph genera | Fixed effect | Estimate | SE | df | t | p |
| --- | --- | --- | --- | --- | --- | --- |
| *Methylacidimicrobium* | Intercept | 3816 | 1426 | 28.94 | 2.676 | **0.01*** |
|  | Methanotroph genera | 266100 | 13030000 | 40.21 | 0.02 | 0.98 |
|  | speciesC.acinaciformis | -839.1 | 1753 | 17.53 | -0.479 | 0.64 |
|  | speciesN.magnus | 1130 | 1770 | 18.11 | 0.638 | 0.5 |
| *Methylobacter* | Intercept | 4026 | 1325 | 22.81 | 3.039 | **0.006*** |
|  | Methanotroph genera | -1E+07 | 25250000 | 41.84 | -0.402 | 0.69 |
|  | speciesC.acinaciformis | -756.8 | 1754 | 17.92 | -0.431 | 0.67 |
|  | speciesN.magnus | 1131 | 1760 | 18.07 | 0.643 | 0.53 |
| *Methylocaldum* | Intercept | 3838 | 1419 | 28.37 | 2.704 | **0.01*** |
|  | Methanotroph genera | -108200 | 10680000 | 40.83 | -0.01 | 0.99 |
|  | speciesC.acinaciformis | -836.9 | 1757 | 17.64 | -0.476 | 0.64 |
|  | speciesN.magnus | 1130 | 1770 | 18.07 | 0.638 | 0.53 |
| *Methylocapsa* | Intercept | 3794.45 | 1247.62 | 17.74 | 3.041 | **0.007*** |
|  | Methanotroph genera | 165119.4 | 439592.2 | 41.34 | 0.376 | 0.71 |
|  | speciesC.acinaciformis | -892.29 | 1765.06 | 17.77 | -0.506 | 0.62 |
|  | speciesN.magnus | 1122.7 | 1776.35 | 18.14 | 0.632 | 0.534 |
| *Methylocella* | Intercept | 3785.87 | 1259.42 | 18.5 | 3.006 | **0.007*** |
|  | Methanotroph genera | 213799.9 | 999328.4 | 41.29 | 0.214 | 0.83 |
|  | speciesC.acinaciformis | -863.23 | 1760.32 | 17.69 | -0.49 | 0.63 |
|  | speciesN.magnus | 1123.38 | 1773.83 | 18.15 | 0.633 | 0.53 |
| *Methylococcus* | Intercept | 4042.57 | 1556.12 | 37.61 | 2.598 | **0.01*** |
|  | Methanotroph genera | -953640 | 4254134 | 39.83 | -0.224 | 0.82 |
|  | speciesC.acinaciformis | -819.63 | 1749.6 | 17.6 | -0.468 | 0.65 |
|  | speciesN.magnus | 1128.31 | 1764.95 | 18.12 | 0.639 | 0.53 |
| *Methylocystis* | Intercept | 3830.74 | 1290.73 | 20.42 | 2.968 | **0.007*** |
|  | Methanotroph genera | -216.34 | 475866.5 | 41.55 | 0 | 1.00 |
|  | speciesC.acinaciformis | -838.06 | 1757.3 | 17.7 | -0.477 | 0.64 |
|  | speciesN.magnus | 1129.87 | 1770.51 | 18.15 | 0.638 | 0.53 |
| *Methylomicrobium* | Intercept | 3928 | 1393 | 27.03 | 2.819 | **0.009*** |
|  | Methanotroph genera | -1641000 | 10820000 | 40.24 | -0.152 | 0.88 |
|  | speciesC.acinaciformis | -817 | 1756 | 17.77 | -0.465 | 0.65 |
|  | speciesN.magnus | 1129 | 1767 | 18.15 | 0.639 | 0.53 |
| *Methylomonas* | Intercept | 3984.84 | 1408.84 | 28.05 | 2.828 | **0.009*** |
|  | Methanotroph genera | -781010 | 3419619 | 40.48 | -0.228 | 0.82 |
|  | speciesC.acinaciformis | -819.41 | 1750.41 | 17.61 | -0.468 | 0.65 |
|  | speciesN.magnus | 1126.76 | 1765.81 | 18.14 | 0.638 | 0.53 |
| *Methylosinus* | Intercept | 3834.32 | 1315.65 | 21.88 | 2.914 | **0.008*** |
|  | Methanotroph genera | -11625.3 | 1367944 | 41.72 | -0.008 | 0.99 |
|  | speciesC.acinaciformis | -837.26 | 1756.09 | 17.66 | -0.477 | 0.64 |
|  | speciesN.magnus | 1129.93 | 1770.4 | 18.15 | 0.638 | 0.53 |

Supplementary Table 9. Model summary of linear mixed effects model testing all relevant predictors of CH_4_ emission to understand variation within mounds. For termite species-level comparisons, *A. laurensis* is the reference species.

| Fixed effects | Estimate | Std. Error | df | t value | p |
| --- | --- | --- | --- | --- | --- |
| intercept | 5470.08 | 1904.12 | 40.64 | 2.87 | **0.006*** |
| pmoA relative abundance | -1225.34 | 678.22 | 32.63 | -1.81 | 0.08 |
| Species - *C. acinaciformis* | 244.10 | 1618.62 | 13.41 | 0.15 | 0.88 |
| Species - *N. magnus* | 3420.91 | 1533.97 | 11.32 | 2.23 | **0.047*** |
| PCoA1 | -13070.41 | 35193.02 | 52.13 | -0.371 | 0.71 |
| PCoA2 | 12518.02 | 30237.22 | 52.51 | 0.41 | 0.68 |
| Relative abundance methanotroph sum | -392888.56 | 771264.09 | 53.64 | -0.51 | 0.61 |
| PCoA1 * PCoA2 | 469113.2 | 601090.41 | 53.34 | -0.78 | 0.43 |

Supplementary Table 10. Summary of published CH_4_ emissions for termite mounds at the species level. Note that the sample sizes from this study include mounds that were a part of the initial, larger sampling campaign, hence the greater sample size for each species.

| Study | Species | n | Mound-level emission  (μmol CH_4_ h^-1^ mound^-1^) | Average total mound volume (m^3^) |
| --- | --- | --- | --- | --- |
| This study  (Australia) | *Amitermes laurensis* | 14 | 57.11 (average) | 0.04 |
|  | *Coptotermes acinaciformis* | 25 | 304.79 (average) | 0.80 |
|  | *Nasutitermes magnus* | 19 | 1290.23 (average) | 0.94 |
| Van Asperen et al. 2021  (Brazilian Amazon) | *Neocapritermes brasiliensis* | 5 | 61-125 (range) | 0.05 |
| Khalil et al., 1990  (Australia) | *Amitermes laurensis* | n/a | 67.39 (average) | n/a |
|  | *Drepanotermes perniger* | 3-5 | 134.78 (average) | n/a |
|  | *Nasutitermes magnus* | 3-5 | 112.32 (average) | n/a |
|  | *Nasutitermes triodae* | 3-5 | 89.86 (average) | n/a |
|  | *Tumulitermes pastinator* | 3-5 | 67.39 (average) | n/a |
|  | *Coptotermes lacteus* | 2 | 8.99 (average) | n/a |
| Macdonald et al., 1998  (Cameroon) | *Bulbitermes sp. C* | 3 | 8.83 (average) | n/a |
|  | *Dicuspiditermes nemorosus* | 6 | 10.91 (average) | n/a |
|  | *Dicuspiditermes santschii* | 12 | 20.69 (average) | n/a |
|  | *Prohamitermes mirabilis* | 2 | 0.32 (average) | n/a |
| Martius et al., 1993  (Brazilian Amazon) | *Nasutitermes ephratae* | 5 | 172.07 (average) | 0.03 |
|  | *Nasutitermes macrocephahts* | 1 | 24.94 | 0.007 |
|  | *Nasutiteones surinamensis* | 2 | 177.68 (average) | 0.03 |
|  | *Nasutitermes comiger* | 1 | 12.47 | 0.001 |
|  | *Nasutitermes araujoi* | 1 | 143.39 | 0.04 |
| Seiler et al., 1984  (South Africa) | *Odontotermes sp.* | 3 | 4.36 (average) | n/a |
|  | *Macrotermes sp.* | 5 | 168.33 (average) | n/a |
|  | *Cubitermes sp.* | 7 | 1.43 (average) | n/a |
|  | *Amitermes sp.* | 17 | 4.99 (average) | n/a |
|  | *Hodotermes sp.* | 3 | 4.30 (average) | n/a |
|  | *Trinervitermes sp.* | 10 | 642.14 (average) | n/a |
| Sugimoto et al., 1998  (Thailand) | *Microcerotermes sp.* | 2 | 1.59 (average) | n/a |
|  | *Globitermes sulphures* | 1 | 1.79 | n/a |
|  | *Termes sp.* | 1 | 0.71 | n/a |
|  | *Dicuspiditermes sp* | 3 | 0.65 (average) | n/a |

Supplementary Table 11. List of bacterial genera associated with methanotrophy. These genera were used to query the metagenomics dataset for methanotroph candidates to include in our analysis on variation in bacterial methanotroph community composition within and between mounds.

| **Genera** | **Reference** |
| --- | --- |
| *Methylobacter* | Zhou et al., 2015 |
| *Methylomonas* | Bowman et al., 1993 |
| *Methylosarcina* | Kalyuzhnaya 2016a |
| *Methylomicrobium* | Kalyuzhnaya 2016b |
| *Methylococcus* | Bowman et al., 1993 |
| *Methylocystis* | Bowman et al., 1993 |
| *Methylosinus* | Bowman et al., 1993 |
| *Methylacidiphilum* | Awala et al., 2023 |
| *Methylacidimicrobium* | van Teeseling et al., 2014 |
| *Methylomirabilis* | Zhu et al., 2022 |
| *Methylocapsa* | Dunfield et al., 2010 |
| *Methylocella* | Dunfield et al., 2010 |
| *Methylohalobius* | Dunfield 2016 |
| *Methylothermus* | Houghton et al., 2019 |
| *Methylosphaera* | Bowman 2015 |
| *Methylocaldum* | Delherbe et al., 2024 |

Supplementary Table 12. Coefficient of Variation (CV) in CH_4_ emissions for individual mounds (calculated from 4-5 emission subsamples) which were remeasured in all four seasons.

| Mound ID and season | Species | CV CH_4_ |
| --- | --- | --- |
| MD10-aug23 | *A. laurensis* | 0.72518751 |
| MD10-feb24 | *A. laurensis* | 0.15649616 |
| MD10-may22 | *A. laurensis* | 0.23420207 |
| MD10-nov22 | *A. laurensis* | 0.62684765 |
| MD11-aug23 | *N. magnus* | 0.13976645 |
| MD11-feb24 | *N. magnus* | 0.43655448 |
| MD11-may22 | *N. magnus* | 0.54947462 |
| MD11-nov22 | *N. magnus* | 0.70120433 |
| MD15-aug23 | *A. laurensis* | 0.71629532 |
| MD15-feb24 | *A. laurensis* | 0.59431377 |
| MD15-may22 | *A. laurensis* | 0.40346158 |
| MD15-nov22 | *A. laurensis* | 0.82795137 |
| MD2-aug23 | *C. acinaciformis* | 0.85894341 |
| MD2-feb24 | *C. acinaciformis* | 0.72069127 |
| MD2-may22 | *C. acinaciformis* | 0.48623917 |
| MD2-nov22 | *C. acinaciformis* | 0.70720512 |
| MD24-aug23 | *A. laurensis* | 0.70530019 |
| MD24-feb24 | *A. laurensis* | 0.80467255 |
| MD24-may22 | *A. laurensis* | 0.71433979 |
| MD24-nov22 | *A. laurensis* | 0.8707335 |
| MD25-aug23 | *N. magnus* | 3.23211397 |
| MD25-feb24 | *N. magnus* | 0.44941179 |
| MD25-may22 | *N. magnus* | 0.38490256 |
| MD25-nov22 | *N. magnus* | 0.6213232 |
| MD26-aug23 | *C. acinaciformis* | 0.44222093 |
| MD26-feb24 | *C. acinaciformis* | 0.89459261 |
| MD26-may22 | *C. acinaciformis* | 0.5766301 |
| MD26-nov22 | *C. acinaciformis* | 0.5172309 |
| MD27-aug23 | *C. acinaciformis* | 0.54180697 |
| MD27-feb24 | *C. acinaciformis* | 0.34956184 |
| MD27-may22 | *C. acinaciformis* | 0.52752155 |
| MD27-nov22 | *C. acinaciformis* | 0.57263334 |
| MD36-aug23 | *A. laurensis* | 0.65603574 |
| MD36-feb24 | *A. laurensis* | 1.74962122 |
| MD36-may22 | *A. laurensis* | 0.1312587 |
| MD36-nov22 | *A. laurensis* | 0.2754898 |
| MD37-aug23 | *A. laurensis* | 0.94895901 |
| MD37-feb24 | *A. laurensis* | 0.81529395 |
| MD37-may22 | *A. laurensis* | 0.42929067 |
| MD37-nov22 | *A. laurensis* | 0.37072937 |
| MD42-aug23 | *A. laurensis* | 1.24871031 |
| MD42-feb24 | *A. laurensis* | 0.41290271 |
| MD42-may22 | *A. laurensis* | 0.28401925 |
| MD42-nov22 | *A. laurensis* | 0.55448877 |
| MD48-aug23 | *C. acinaciformis* | 1.24964188 |
| MD48-feb24 | *C. acinaciformis* | 1.67121951 |
| MD48-may22 | *C. acinaciformis* | 0.54201543 |
| MD48-nov22 | *C. acinaciformis* | 1.41279478 |
| MD50-aug23 | *N. magnus* | 1.53901682 |
| MD50-feb24 | *N. magnus* | 0.88916677 |
| MD50-may22 | *N. magnus* | 0.45238967 |
| MD50-nov22 | *N. magnus* | 0.6003314 |
| MD54-aug23 | *A. laurensis* | 0.68330327 |
| MD54-feb24 | *A. laurensis* | 0.73809884 |
| MD54-may22 | *A. laurensis* | 0.27329997 |
| MD54-nov22 | *A. laurensis* | 0.57514925 |
| MD55-aug23 | *C. acinaciformis* | 0.5431953 |
| MD55-feb24 | *C. acinaciformis* | 0.44350083 |
| MD55-may22 | *C. acinaciformis* | 0.17702524 |
| MD55-nov22 | *C. acinaciformis* | 1.43493869 |
| MD58-aug23 | *N. magnus* | 0.56719051 |
| MD58-feb24 | *N. magnus* | 0.49223823 |
| MD58-may22 | *N. magnus* | 0.66090335 |
| MD58-nov22 | *N. magnus* | 0.53890282 |
| MD59-aug23 | *C. acinaciformis* | 0.73365633 |
| MD59-feb24 | *C. acinaciformis* | 0.93867452 |
| MD59-may22 | *C. acinaciformis* | 0.8238158 |
| MD59-nov22 | *C. acinaciformis* | 0.62781148 |
| MD63-aug23 | *C. acinaciformis* | 15.4815554 |
| MD63-feb24 | *C. acinaciformis* | 1.26466102 |
| MD63-may22 | *C. acinaciformis* | 0.84613584 |
| MD63-nov22 | *C. acinaciformis* | 1.01720502 |
| MD65-aug23 | *N. magnus* | 0.41842549 |
| MD65-feb24 | *N. magnus* | 0.80182628 |
| MD65-may22 | *N. magnus* | 0.48453621 |
| MD65-nov22 | *N. magnus* | 0.46921532 |

Supplementary Table 13. Mean mound size (as surface area, m^2^ and as volume, m^3^) and mound wall thickness (mm) for the three study species.

| Species | Mean mound surface area (m^3^) | Mean mound volume (m^3^) | Mean mound wall thickness (mm) |
| --- | --- | --- | --- |
| *A. laurensis* | 0.7 | 0.04 | 7.80 |
| *C. acinaciformis* | 4.1 | 0.78 | 73.93 |
| *N. magnus* | 5.5 | 0.94 | 12.93 |

References

Awala, S. I., Gwak, J. H., Kim, Y., Seo, C., Strazzulli, A., Kim, S. G., & Rhee, S. K. (2023). Methylacidiphilum caldifontis gen. Nov., sp. Nov., a thermoacidophilic methane-oxidizing bacterium from an acidic geothermal environment, and descriptions of the family Methylacidiphilaceae fam. Nov. And order Methylacidiphilales ord. Nov. International Journal of Systematic and Evolutionary Microbiology, 73(10), 006085. <https://doi.org/10.1099/ijsem.0.006085>

Bowman, J.P. (2015). Methylosphaera. In Bergey's Manual of Systematics of Archaea and Bacteria (eds M.E. Trujillo, S. Dedysh, P. DeVos, B. Hedlund, P. Kämpfer, F.A. Rainey and W.B. Whitman). <https://doi.org/10.1002/9781118960608.gbm01185>

Bowman, J. P., Sly, L. I., Nichols, P. D., & Hayward, A. C. (1993). Revised Taxonomy of the Methanotrophs: Description of Methylobacter gen. nov., Emendation of Methylococcus, Validation of Methylosinus and Methylocystis Species, and a Proposal that the Family Methylococcaceae Includes Only the Group I Methanotrophs. International Journal of Systematic and Evolutionary Microbiology, 43(4), 735–753. <https://doi.org/10.1099/00207713-43-4-735>

Clement, R. A., Flores-Moreno, H., Cernusak, L. A., Cheesman, A. W., Yatsko, A. R., Allison, S. D., Eggleton, P., & Zanne, A. E. (2021). Assessing the Australian Termite Diversity Anomaly: How Habitat and Rainfall Affect Termite Assemblages. *Frontiers in Ecology and Evolution*, *9*, 657444.<https://doi.org/10.3389/fevo.2021.657444>

Delherbe, N. A., Pearce, D., But, S. Y., Murrell, J. C., Khmelenina, V. N., & Kalyuzhnaya, M. G. (2024). Genomic Insights into Moderately Thermophilic Methanotrophs of the Genus *Methylocaldum*. *Microorganisms*, 12(3), 469. https://doi.org/10.3390/microorganisms12030469

Dunfield, P. F., Belova, S. E., Vorob’ev, A. V., Cornish, S. L., & Dedysh, S. N. (2010). Methylocapsa aurea sp. Nov., a facultative methanotroph possessing a particulate methane monooxygenase, and emended description of the genus Methylocapsa. International Journal of Systematic and Evolutionary Microbiology, 60(11), 2659–2664. <https://doi.org/10.1099/ijs.0.020149-0>

Dunfield, P.F. (2016). Methylohalobius. In Bergey's Manual of Systematics of Archaea and Bacteria (eds M.E. Trujillo, S. Dedysh, P. DeVos, B. Hedlund, P. Kämpfer, F.A. Rainey and W.B. Whitman). <https://doi.org/10.1002/9781118960608.gbm01418>

Houghton, K. M., Carere, C. R., Stott, M. B., & McDonald, I. R. (2019). Thermophilic methanotrophs: In hot pursuit. *FEMS Microbiology Ecology*, 95(9), fiz125. <https://doi.org/10.1093/femsec/fiz125>

Kalyuzhnaya, M.G. (2016a). *Methylosarcina*. Bergey's Manual of Systematics of Archaea and Bacteria (eds W.B. Whitman, F. Rainey, P. Kämpfer, M. Trujillo, J. Chun, P. DeVos, B. Hedlund and S. Dedysh). <https://doi.org/10.1002/9781118960608.gbm01184.pub2>

Kalyuzhnaya, M.G. (2016b). *Methylomicrobium*. In Bergey's Manual of Systematics of Archaea and Bacteria (eds W.B. Whitman, F. Rainey, P. Kämpfer, M. Trujillo, J. Chun, P. DeVos, B. Hedlund and S. Dedysh). <https://doi.org/10.1002/9781118960608.gbm01182.pub2>

Khalil, M., Rasmussen, R., French, J. R., & Holt, J. A. (1990). The Influence of Termites on Atmospheric Trace Gases: CH4, CO2, CHC13, N20, CO, H2, and Light Hydrocarbon. *Journal of Geophysical Research*, *95*, 3619–3634.

Macdonald, J. A., Eggleton, P., Bignell, D. E., Forzi, F., & Fowler, D. (1998). Methane emission by termites and oxidation by soils, across a forest disturbance gradient in the Mbalmayo Forest Reserve, Cameroon. *Global Change Biology*, *4*(4), 409–418.<https://doi.org/10.1046/j.1365-2486.1998.00163.x>

Martius, C., Wassmann, R., Thein, U., Bandeira, A., Rennenberg, H., Junk, W., & Seiler, W. (1993). Methane emission from wood-feeding termites in Amazonia. *Chemosphere*, *26*(1), 623–632.<https://doi.org/10.1016/0045-6535(93)90448-E>

Seiler, W., Conrad, R., & Scharffe, D. (1984). Field studies of methane emission from termite nests into the atmosphere and measurements of methane uptake by tropical soils. *Journal of Atmospheric Chemistry*, *1*(2), 171–186.<https://doi.org/10.1007/BF00053839>

Sparks, A. (2018). “nasapower: A NASA POWER Global Meteorology, Surface Solar Energy and Climatology Data Client for R.” The Journal of Open Source Software, 3(30), 1035. doi:10.21105/joss.01035.

Sugimoto, A., Inoue, T., Kirtibutr, N., & Abe, T. (1998). Methane oxidation by termite mounds estimated by the carbon isotopic composition of methane. *Global Biogeochemical Cycles*, *12*(4), 595–605.<https://doi.org/10.1029/98GB02266>

Van Asperen, H., Alves-Oliveira, J. R., Warneke, T., Forsberg, B., Carioca De Araújo, A., & Notholt, J. (2021). The role of termite CH4 emissions on the ecosystem scale: A case study in the Amazon rainforest. *Biogeosciences*, *18*(8), 2609–2625.<https://doi.org/10.5194/bg-18-2609-2021>

Van Teeseling M. C. F, Pol A., Harhangi H. R., van der Zwart S., Jetten M. S. M, Op den Camp H. J. M., van Niftrik L. (2014). Expanding the Verrucomicrobial Methanotrophic World: Description of Three Novel Species of Methylacidimicrobium gen. nov. *Appl Environ Microbiol,* 80(21). <https://doi.org/10.1128/AEM.01838-14>.

Zhu B., Karwautz C., Andrei S., Klingl A., Pernthaler J., Lueders T. (2022). A novel *Methylomirabilota* methanotroph potentially couples methane oxidation to iodate reduction. *mLife*, 1, 323–328. <https://doi.org/10.1002/mlf2.12033>
